# Expression of Subtelomeric lncRNAs Links Telomeres Dynamics to RNA Decay in *S. cerevisiae*

**DOI:** 10.3390/ncrna1020094

**Published:** 2015-07-03

**Authors:** Marta Kwapisz, Myriam Ruault, Erwin van Dijk, Stephanie Gourvennec, Marc Descrimes, Angela Taddei, Antonin Morillon

**Affiliations:** 1 ncRNA, epigenetics and genome fluidity, Institut Curie, PSL Research University, CNRS UMR3244, Université Pierre et Marie Curie, 26 rue d’Ulm, 75248 Paris Cedex 05, France; E-Mail: marc.descrimes@curie.fr (M.D.); 2 Nuclear Dynamics, Institut Curie, PSL Research University, CNRS UMR3664, Université Pierre et Marie Curie, 26 rue d’Ulm, 75248 Paris Cedex 05, France; E-Mails: myriam.ruault@curie.fr (M.R.); angela.taddei@curie.fr (A.T.)

**Keywords:** subtelomeric region, non-coding RNA, telomere maintenance, *S. cerevisiae*, Rap1p

## Abstract

Long non-coding RNAs (lncRNAs) have been shown to regulate gene expression, chromatin domains and chromosome stability in eukaryotic cells. Recent observations have reported the existence of telomeric repeats containing long ncRNAs – *TERRA* in mammalian and yeast cells. However, their functions remain poorly characterized. Here, we report the existence in *S. cerevisiae* of several lncRNAs within Y′ subtelomeric regions. We have called them *subTERRA*. These belong to Cryptic Unstable Transcripts (CUTs) and Xrn1p-sensitive Unstable Transcripts (XUTs) family. *subTERRA* transcription, carried out mainly by RNAPII, is initiated within the subtelomeric Y’ element and occurs in both directions, towards telomeres as well as centromeres. We show that *subTERRA* are distinct from *TERRA* and are mainly degraded by the general cytoplasmic and nuclear 5′- and 3′- RNA decay pathways in a transcription-dependent manner. *subTERRA* accumulates preferentially during the G1/S transition and in C-terminal *rap1* mutant but independently of Rap1p function in silencing. The accumulation of *subTERRA* in RNA decay mutants coincides with telomere misregulation: shortening of telomeres, loss of telomeric clustering in mitotic cells and changes in silencing of subtelomeric regions. Our data suggest that subtelomeric RNAs expression links telomere maintenance to RNA degradation pathways.

## 1. Introduction

In *Saccharomyces cerevisiae*, telomeres consist of 350 ± 75 base pairs of C_1–3_A/TG_1–3_ DNA and are required for the stable maintenance of chromosomes [1]. This size is kept within a narrow size range, specific for a particular strain and does not change in culture [2]. Simple telomeric C_1-3_A/TG_1–3_ repeats are followed by two subtelomeric sequences: the X-core and the Y′ [3]. X-core elements (around 300 bp) are present in all telomeres and Y′ (long of 6.7 kb or short of 5.2 kb) in about 70% of telomeres. Y′ appears in 1 to 4 tandem copies separated by short tracks of telomeric repeats. X as well as Y′ sequences differ among telomeres by short, multiple insertions and/or deletions [4,5,6]; In consequence, subtelomeric regions are heterogeneous structures that vary between different cells of a strain as well as from strain to strain [7]. These variations encourage differences in protein factors bound to the region thereby [8] causing diversity at the level of chromatin structure and transcriptional activity at individual chromosome ends [9]. In general, subtelomeric elements impose transcriptional repression of nearby sequences (TPE – Telomere Position Effect; [10,11];) and create hetero-euchromatic boundaries as insulators or poised promoters [12]. The X core is transcriptionally silent, has low histone content with hypoacetylated histone H4 (H4K16) while Y′ elements, particularly distal ones, are rather euchromatic. The role of Y′ elements and more largely subtelomeric regions in telomere maintenance is not clear.

Subtelomeres and telomeres are dynamic sequences undergoing recurrent recombination and shortening. Their maintenance in the cell is in equilibrium between synthesis and erosion. Telomeres are late and slow replicating [13], which results from position effects exerted by telomeres on proximal replication origins and depends on telomere length [14]. Each replication event results in telomere shortening because of an inherent inability of the replication machinery to fully replicate them [15]. This sequence loss is normally prevented by the action of the ribonucleoprotein enzyme telomerase, which reverse-transcribes telomeric repeats onto telomeric ends [16]. Telomerase-deficient cells enter into crisis and early senescence. However, a small fraction of cells can survive and maintain telomeric DNA through recombination [17]. These survivors, also called ALTs (Alternative Lengthening of Telomeres; [18]), form through two mechanisms; type I (ALT1) contains multiple tandem Y’ elements that arise predominantly through replication-dependent recombination [17]; and type II (ALT2), probably lengthen TG_1-3_ tracks *via* rolling circle replication [19] and exhibit an increase in telomeric repeats [18]. Telomere maintenance by recombination is widespread occurring from yeast to mammals [17] and approximately 10% to 15% of human cancers are immortalized due to ALT conversion [20].

Telomeric repeats are recognized by the essential DNA-binding protein Rap1p (Repressor Activator Protein 1; ([21]). Rap1p serves as a platform for different complexes. Rap1p-interacting factors - Rif1p and Rif2p bind to the C-terminal domain and inhibit telomerase activity and telomere lengthening [22]. The C-terminal domain is also recognized by the silencing regulators Sir3p and Sir4p [23], which recruit the NAD-dependent histone deacetylase Sir2p [24,25]. Following Sir2p action, hypoacetylated histones H3 and H4 within the region, become excellent substrates for tethering by silencing factors Sir3p and Sir4p. This leads to spreading of the SIR complex and transcriptional repression of the subtelomeric regions [26,27]. SIRs are restricted to heterochromatin by the activities of histone methyltransferases Set1p and Dot1p [28,29]. Sequestration of SIRs onto telomeres favors both subtelomeric repression and impacts a subset of promoters throughout the genome [30]. Yeast telomeres preferentially cluster to form telomeric foci, which remain associated with the nuclear envelope [31]. Telomere tethering to the nuclear membrane is cell cycle-dependent and requires Sir4, Rap1, yKu, Esc1/2 proteins and the nuclear pore subunits [32,33,34], but *trans* telomere interactions depend only on Sir3p [35]. The amount of Sir3p specifically determines nuclear distribution and dynamics of telomere clusters, which result from random motion, aggregation, and dissociation of telomeric regions [36]. Chromosome arm length and nuclear constraints (nuclear envelope, cell volume and attachment of centromeres to Spindle Pole Body – SPB) are major determinants of transient subtelomere associations [37].

Eukaryotic telomeres are transcribed into telomeric repeat-containing RNA (*TERRA*; [38,39]) that have been reported in mammals, birds, zebra fish, plants, budding and fission yeast *(Saccharomyces cerevisiae* [40]; *Schizosaccharomyces pombe* [41]). Chromosome ends produce distinct RNA species: G-rich *TERRA* transcripts and *ARRET*, subtelomeric RNA species transcribed in the opposite direction of *TERRA*. Moreover, fission yeast telomeres generate C-rich telomeric repeat-containing transcripts (*ARIA*) and subtelomeric transcripts antisense to *ARRET* called *αARRET* [41]. In budding yeast, transcription and degradation of *TERRA* depends on the type of subtelomere element where *TERRA*’s promoter is embedded [42] *i.e.*, *TERRA* is negatively regulated at Y′ telomeres primarily by the Rap1p-binding proteins Rif1/2, with the Sir2/3/4 histone deacetylase complex playing a minor, repressive role. At X-only telomeres both the Sir2/3/4 complex as well as the Rif1/2 proteins are important for promoting *TERRA* repression [42]. Recent data suggest that these RNAs might be a part of the telomeric structure *i.e.*, *TERRA* remain associated with telomeres after its transcription, and SMG proteins (nonsense mediated mRNA decay factors), [39] regulate this association. Furthermore, telomeric transcripts could play a role in the maintenance of telomere structure and heterochromatin formation. In mammalian cells *TERRA* interacts with telomere-associated proteins, including Telomere Repeat Factors 1 and 2 (TRF1/2), subunits of the Origin Recognition Complex (ORC), Heterochromatin Protein 1 (HP1), trimethylated K9 of histone H3 and members of the DNA damage sensing pathway [43]. Together these data suggest that telomeric transcripts, or a subpopulation of these, are regulatory non-coding RNAs.

With the recent discovery of regulatory ncRNAs controlling *cis* and *trans* gene silencing in budding yeast [44,45,46,47], it is tempting to speculate that regulatory ncRNAs are directly involved in heterochromatin regulation at the telomeric regions. Recent reports reveal that RNA-processing proteins *i.e.*, Trf4, Rrp6 and Xrn1 are engaged in the maintenance of genome integrity. They do so indirectly by regulating gene expression at transcriptional and post-transcriptional levels following DNA damage (alternative polyadenylation, rapid degradation, export) and more directly by preventing RNA:DNA hybrid formation [48] or regulating Mec1p signaling activity by promoting formation of RPA(Replication Protein A)-coated ssDNA at DSB (DNA double-strand breaks) ends [49]. In *S. cerevisiae*, the alternative transcription termination complex Nrd1–Nab3 and the exosome subunit Rrp6p are required to maintain heterochromatin stability and silencing at telomeres, suggesting that RNA degradation and transcriptional regulation might control heterochromatinization [50,51].

Here we report the characterization of subtelomeric *subTERRA* lncRNA and ask whether they can control some aspects of the telomere maintenance in yeast *S. cerevisiae*. Our findings reveal that mutations affecting *subTERRA* accumulation correlate with telomere misclustering and change Telomere Position Effects (TPE). We propose that subtelomeric lncRNAs are additional actors in telomere control.

## 2. Results

### 2.1. Characterization of the Subtelomeric Y′ ncRNAs (subTERRA)

**Figure 1:**
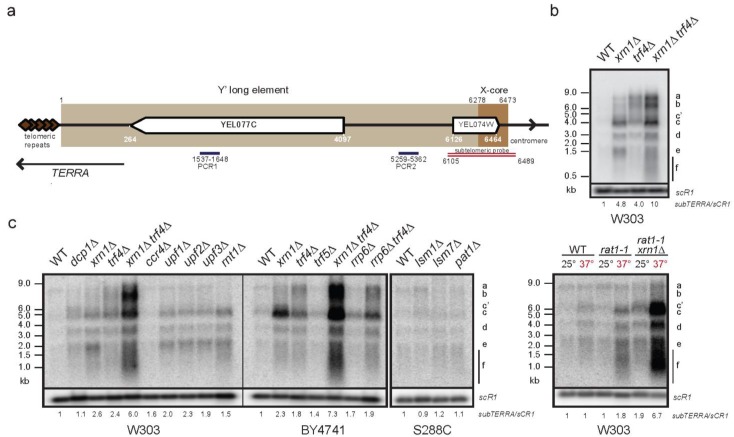
Subtelomeric regions are transcribed yielding unstable Y′ ncRNAs. (**a**) Schematic representation of VL chromosome end with coordinates. Telomeric repeats (around 300 bp) are brown diamonds; subtelomeric long Y′ element (6278 bp) containing putative Y′-helicase gene (*YEL077C*) is light brown; subtelomeric X element core (195 bp) is shown in darker brown. Position of specific subtelomeric double strand probe [51] used for Y’ ncRNA detection is shown in red. PCR1 and PCR2 are oligonucleotide pairs used for *subTERRA* quantification with qPCR; (**b**) Characterization of unstable Y′ RNAs transcribed from subtelomeric regions. Total RNA from WT and mutant strains (W303 genetic background, exponentially grown in rich media (YPD – Yeast Extract, Peptone, Dextrose; at 30 °C) were extracted and analyzed by Northern blot. Signals of Y′ RNAs detected with radiolabelled subtelomeric probe (as shown in 1a) were normalized with the *scR1* RNA level. *scR1* is small cytoplasmic RNA, RNA component of the Signal Recognition Particle (SRP) synthesized by RNAPIII. Representative experiments from at least 10 biological replicates. Signal quantification is shown below respective lines (*subTERRA/scR1*); (**c**) Detection of Y’ RNAs in strains mutated for genes implicated in RNA degradation. Genetic backgrounds are marked under the panels; detection and normalization as in 1b, at minimum, biological duplicates were made. Cells were grown in YPD at 30 °C ON, for the *rat1-1* mutant, cells were grown at 25 °C and shifted to 37 °C for 3h. Signal quantification is shown below respective lines (*subTERRA/scR1*).

In *S. cerevisiae*, promoters within subtelomeric regions give rise to two types of cryptic lncRNAs: *TERRA* [40,42]; a population of 0.2–1.0 kb-long transcripts containing telomeric repeats and *TEL05L* ncRNA or *ARRET* transcribed in opposite direction, both subjected to the nuclear 5′end decay mediated by Rat1p [40,51]. This indicates that subtelomeric regions, considered as silent, might undergo intense transcription yielding a variety of ncRNAs. To characterize these potentially unstable ncRNAs, total RNA from wild type strains and strains defective for cytoplasmic and nuclear RNA decay pathways, were extracted and analyzed by Northern blot. For detection we used a Y′-specific, subtelomeric probe (as in [51]; schematized in Figure 1a) and signals were normalized to *scR1* RNA levels.

In the strain defective for the cytoplasmic 5′–3′ exoribonuclease Xrn1p, we observed an accumulation of three abundant Y′ ncRNA species (labeled c, d, e) ranging from 0.5 to 4 kb and three less abundant ncRNAs, labeled a, b and c’, size ranging between 6 and 9 kb (Figure 1b, 4.8-fold of WT level). This accumulation was dependent on the catalytic activity of Xrn1p since the *xrn1* catalytic mutant exhibited the same phenotype as *xrn1*∆ (Figure S1a). We therefore consider these ncRNAs as novel members of the family of Xrn1p-sensitive Unstable Transcripts (XUTs) [47]. In addition to *xrn1*∆, similar Y’-XUTs can be detected, albeit to a lesser extent in other mutants of cytoplasmic RNA decay (Figure 1c) *i.e.*, *rnt1*∆ mutant lacking nuclear endoribonuclease III, Rnt1p [52]; *upf1*∆*, upf2*∆ and *upf3*∆*,* affected for the Nonsense-Mediated Decay (NMD) [53] and *dcp1*∆ defective for decapping [54]. Interestingly, the strain defective for the 5′–3′ nuclear exoribonuclease Rat1p (*rat1-1*) accumulated, albeit at very low level, the same family of Y′-XUTs. However, the double mutant *rat1-1xrn1*∆ showed additive phenotype (comparable with *xrn1*∆*trf4*∆) but specific for Xrn1p-dependent Y′ transcripts.

On the other hand, the nuclear 3′–5′ decay targeted longer Y′ ncRNAs, as *trf4*∆, lacking poly(A) polymerase of the TRAMP complex, showed accumulation of at least three main RNA species labeled a, b and c. The *trf4-236* mutant affecting poly(A) polymerase activity [55] did not completely recapitulate the deletion phenotype, indicating that another activity of Trf4p could be implicated in production of Y′ ncRNA (Figure S1a). Double mutant *xrn1*∆ *trf4*∆ that affects both cytoplasmic and nuclear degradation pathways, accumulated high levels of each Y′ ncRNAs species (a, b, c, c’, d and e). Furthermore, single deletion of the catalytic subunit of the exosome complex *rrp6*∆ mutant, stabilizing Cryptic Unstable Transcripts, CUTs [56], showed only weak accumulation of class c and *rrp6*∆ *trf4*∆ double mutant showed no additional effect on *trf4*-dependent Y′ ncRNAs (Figure 1c). Further treatments of the samples with RNaseA and DNase I showed that the Y′-specific probe highlighted RNAs only, without any trace of DNA contamination (Figure S1c).

These data show that at least two major classes of Y′ ncRNA are produced from subtelomeric regions. Xrn1p supports degradation of Y’-XUTs probably in the cytoplasm and Y′-CUTs are subject to Trf4p-dependent degradation, most likely in the nucleus [56,57].

Probes at the junction of telomeric repeats and subtelomeric Y′ region (Figure S2a) failed to detect cryptic RNAs in cells defective for RNA decay pathways suggesting that sense and antisense cryptic Y′ RNAs do not overlap within the distal telomeric repeats and are distinct from the *TERRA* RNA (Figures S1b and S2a). Furthermore, *TERRA* is mainly degraded by the nuclear Rat1p-dependent RNA decay and insensitive to Xrn1p as previously shown [40]. Therefore, to distinguish both species, we named these subtelomeric transcripts “*subTERRA”.* Finally, *subTERRA* characterization showed that these transcripts are RNA polymerase (RNAP)II-dependent and partially polyadenylated (about 30%; Figure S1d and e, respectively).

It is important to note that subtelomeric probes used for detection of *subTERRA* impose important limits; they allow detection of transcripts containing 384 nt-long sequence (Figure 1a) but cannot distinguish between RNAs transcribed towards telomeres or centromeres. Subtelomeric regions are difficult to analyze because of repeated character of their sequences. In order to detect *subTERRA* transcripts in a sense-specific manner we have tried numerous oligonucleotide probes covering the Y’-region but many of them showed cross-hybridization (non-specific signal) or no signal at all. To obtain insights into the orientation of *subTERRA*, we performed RT-qPCR using strand-specific primers (Figure S2b). This shows that *subTERRA* transcripts are produced by sense and antisense transcription both sensitive to Xrn1p and Trf4p RNA decay pathways to different extents. It should be also noted that the *subTERRA* steady-state level is sensitive to growth conditions, temperature and genetic backgrounds. We think that this is a common property of lncRNAs and quantification is only indicative for a tendency rather than an absolute value.

Our data demonstrate that subtelomeric regions in yeast are transcribed into heterogeneous, mostly unstable transcripts called *subTERRA.* A strong accumulation of *subTERRA* in mutants for both cytoplasmic and nuclear RNA decay indicates that these two RNA degradation pathways are implicated in *subTERRA* degradation.

### 2.2. subTERRA-XUTs are Transcribed toward Telomeres and subTERRA-CUTs toward Centromeres

**Figure 2:**
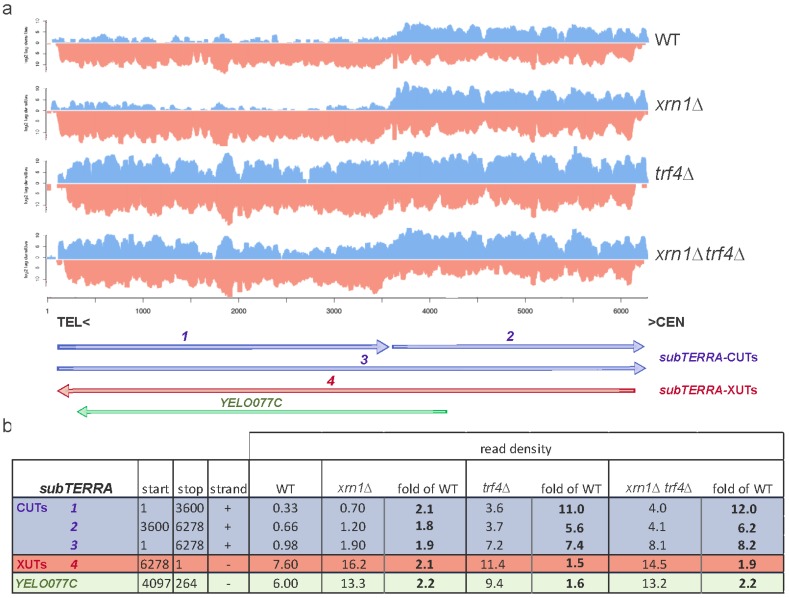
Subtelomeric regions are transcribed into two population of *subTERRA:* CUTs and XUTs. (**a**) Visualization of normalized RNA-seq data at subtelomeric region from position 1 (TG_1–3_ repeats) toward the centromere (6000 bp) for WT, *xrn1*∆*,* *trf4*∆ and *xrn1*∆ *trf4*∆ strains (BY4741). WT and *xrn1*∆ strains data sets are already published ([47]; biological triplicates), *trf4*∆ and *xrn1*∆ *trf4*∆ one culture of each strain was sequenced. *subTERRA* transcripts (*subTERRA*-CUTs, in blue) at positive strand are noted 1–3 and antisense transcript 4 (*subTERRA*-XUTs, in red); Y′-Help1 helicase [58] is noted as *YEL077C* (in green). RNA quantities are expressed as log_2_ tag/read densities; (**b**) Quantification of normalized data for each identified *subTERRA* transcript and calculated fold enrichment over WT level.

To precisely determine the transcriptional landscape of subtelomeric regions, RNA-seq experiments were performed for *trf4*∆ and *xrn1∆* *trf4*∆, and compared with already published WT and *xrn1∆* RNA-seq data [47]. As observed with Northern blot experiments, different classes of *subTERRA* could be defined. Their positions and expression levels were measured as read densities and detailed in Figure 2a,b, respectively. Y′ repetitive sequence are represented as one reference genome and not reflecting each of the repeat. Our mapping cannot discriminate between all the repeats. On this artificial “Y′ genome” we have represented the segments generated by segmentation process. We have identified 4 different RNA species in both directions (species 1–4 at Figure 2a).

In the WT strain all species of *subTERRA* are detectable but at different levels. *subTERRA-*CUT-1 is present at very low levels but *subTERRA*-XUT-4 and *subTERRA-*CUTs-2 and -3 are quite abundant.

Quantity of *subTERRA*-CUTs is strongly increased in *trf4*∆ mutants (11-fold for transcripts 1 and 5.6 to 7.6-fold for transcripts 2 and 3; Figure 2b). *subTERRA*-XUTs are mostly transcribed toward telomeres, they are already present at high level in the WT strain but still accumulate in *xrn1*∆ strain (2.1-fold increase; Figure 2b). There is a moderate 1.5/1.9-fold enrichment of *subTERRA*-CUTs and *subTERRA*-XUTs in *xrn1*∆ and *trf4*∆, respectively.

Both RNA-seq and Northern blot data show that subtelomeric regions are transcribed in both orientations and give rise to a heterogeneous set of transcripts. The clear separation between two types of subtelomeric RNAs, depending potentially on their degradation pathways, argues that the directionality of transcription determines the fate of these RNAs and implies that different subtelomeric RNAs could, in a dosage-dependent manner, have independent functions and cellular localization.

### 2.3. subTERRA Level is Mainly Regulated Post-Transcriptionally

**Figure 3:**
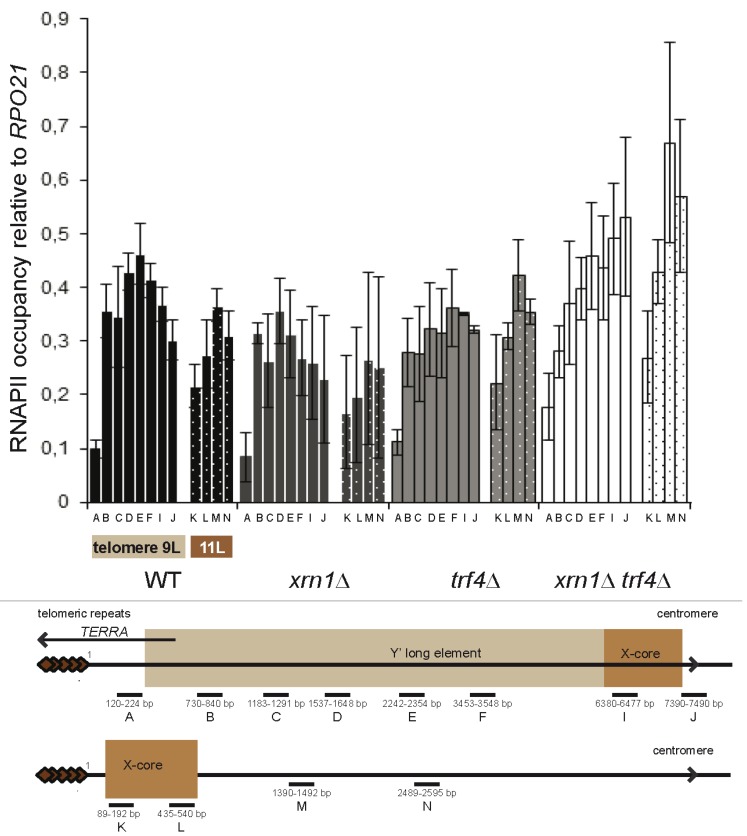
RNAPII occupancy of subtelomeric regions does not change in RNA decay mutants. RNAPII-ChIP experiment in YPD exponentially grown WT, *xrn1*∆ *trf4*∆ and *xrn1*∆ *trf4*∆ strains (W303) scanning subtelomeric region at telomeres 9L (Y′) and 11L (only X). Biological triplicate, RNAPII occupancy was normalized to levels at *RPO21* locus. Pairs of primers used for qPCR are named with letters and represented on schematic view of analyzed regions.

To address the level at which *subTERRA* is regulated, we asked whether high levels of steady-state *subTERRA* in RNA decay pathways mutants are due to RNA synthesis or degradation. To distinguish between these possibilities, we performed RNAPII-ChIP experiments scanning subtelomeric regions (Figure 3). Cross-linked chromatin was extracted from wild type strain and RNA decay mutants *xrn1*∆ *trf4*∆ and *xrn1*∆ *trf4*∆. RNAPII levels were measured by qPCR and normalized with RNAPII quantity at the *RPO21* gene. Figure 3 shows that RNAPII occupancy remained constant in the tested mutants, suggesting that the main regulation of *subTERRA* is post-transcriptional. That was confirmed in *alpha-*factor-synchronized cells, arrested in G1 phase when *subTERRA* levels increased but not RNAPII (at START point G1/S transition, at 1N DNA content; Figure S3). These findings support that *subTERRA* levels are mainly controlled by RNA degradation activities.

### 2.4. subTERRA Localizes to the Nucleus

**Figure 4:**
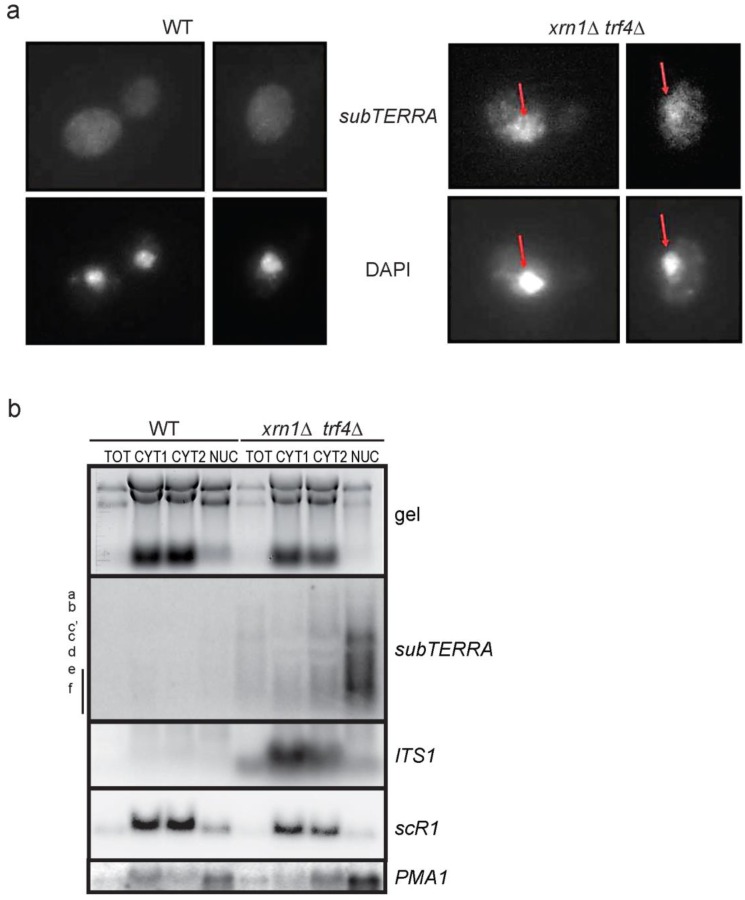
*subTERRA* localize to the nucleus. **(a)** FISH with labeled probes detection of Y′ subtelomeric region (upper panel) and DAPI staining for nuclear DNA detection. Representative cells for WT (W303) strain with no *subTERRA*-specific signals (experiment was representative for biological triplicate). In *xrn1*∆ *trf4*∆ specific *subTERRA* signals co-localize with DAPI staining; **(b)** *subTERRA* were detected almost exclusively in nuclear fraction. Cellular fractionation experiment (biological duplicates): whole RNA extracts (TOT 1/100 of material used for fractionating), two cytoplasmic fractions (CYT1 and CYT2) and nuclear fraction (NUC) were separated on 1% formaldehyde/MOPS agarose gel (upper panel) rRNA and tRNA species are stained with ethidium bromide. RNAs were transferred to nylon membrane and indicated ncRNAs were detected with specific radio-labeled probes (oligonucleotides/single-stranded for *ITS1*, *PMA1*, *scR1* or double-stranded for *subTERRA*).

We investigated the cellular localization of *subTERRA* by performing RNA Fluorescence *In Situ* Hybridization (RNA-FISH) experiments in *xrn1*∆ *trf4*∆ and *xrn1*∆ *trf4*∆ strains accumulating high levels of *subTERRA*. Using subtelomere-specific probes, we observed signals forming a circular shape, co-localizing with DAPI staining (Figure 4a). This indicated that *subTERRA* localized mainly to the nucleus, with a fraction still present in the cytoplasm. To confirm this observation, cell extracts were fractionated and RNAs analyzed by Northern blot. Very low levels of *subTERRA* were detected in any fraction of WT cells, confirming that *subTERRA* is highly sensitive to RNA decay pathways. In *xrn1*∆ *trf4*∆ strain, *subTERRA* were substantially detected in the nuclear fraction with only low quantity detected in the cytoplasm. In contrast, *ITS1* and *scR1* RNAs (expected to be cytoplasmic; [59]) were found in the cytoplasmic fraction (Figure 4b).

Altogether, these data strongly indicate that *subTERRA* localize to the nucleus and might play a role in telomere maintenance through their transcription or through direct action of RNA molecules.

### 2.5. subTERRA Accumulates in rap1-17 Mutant

Since *subTERRA* level is mainly regulated post-transcriptionally, we asked which factors, associated with telomeres, could be implicated in regulation of *subTERRA* expression. To simplify, telomere associated factors could be divided into two groups; structural factors (Rif1/2p, Esc1p, yKu70/80p, Rap1p, telomerase Est1/2/3p and *TLC1*-RNA) and chromatin regulators (such as Set1, Dot1, SIR proteins). Using Northern blot we studied the effect of deletion of genes from both groups on *subTERRA* accumulation. Tested mutants showed very slight or no effect, depending on the genetic background (Figure S4). The phenotype does not exhibit any additional change when combined with *xrn1*∆ mutation.

**Figure 5:**
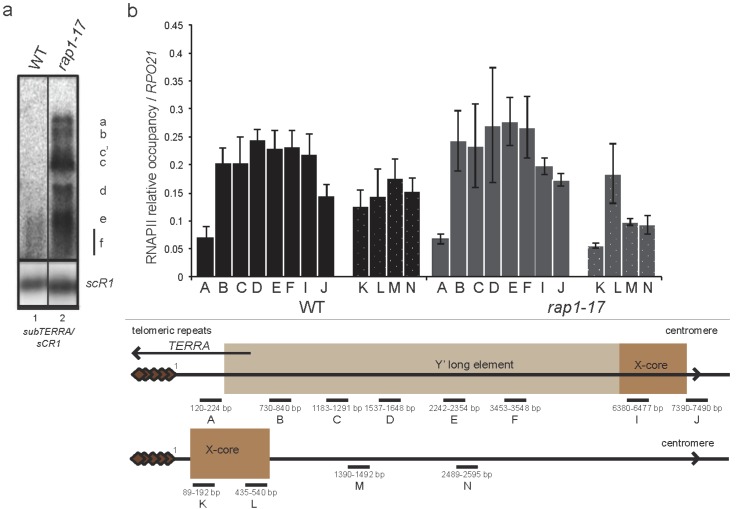
Rap1p regulates *subTERRA* expression at post-transcriptional level. (**a**) Northern blot experiment: total RNA from wild type and *rap1-17* mutant strains grown in YPD at 30 °C were extracted and analyzed by Northern blot. Signals of *subTERRA* detected with radio-labeled subtelomeric probe (as shown in 1a) were normalized with the *scR1* RNA level, at least biological triplicate. *rap1-17* strain accumulated strongly all *subTERRA* species; (**b**) RNAPII-ChIP experiment in YPD exponentially grown WT and *rap1-17* strains scanning subtelomeric region at telomeres 9L and 11L. Biological triplicate, RNAPII occupancy was normalized to levels at *RPO21* locus. Pairs of primers used for qPCR are named with letters and represented on schematic view of analyzed region. Sequences are provided in the Material and Methods Section.

Strikingly, only the *rap1-17* mutation led to an increase in *subTERRA* steady-state level (Figure 5a and Figure S1b). Rap1p is an essential transcription-activation factor [60], which binds telomeric repeats and recruits SIR factors and Rif1/2p at chromosome ends [22,23]. Regarding its crucial role in regulation of protein composition at telomeres, Rap1p impacts telomere length (through recruitment of Rif1/2p) and TPE (SIR’s recruitment). It has been also shown that Rap1p (in *rap1-17* mutant) plays an important role in *TERRA* transcription [42]. In our experiments we have used *rap1-17* allele resulting in production of C-terminal-truncated version of Rap1p, which could not interact with Sir3/4 and Rif1/2 proteins. However, our data suggests that Sir3/4p and Rif1/2p might be involved in regulation of *subTERRA* expression. Combined mutations of *sir3*∆, *sir4*∆ and *rif1/2*∆ did not recapitulate *rap1-17* phenotype, suggesting an alternative function for the C-terminal domain of Rap1p in regulating *subTERRA* levels (Figure S4). We analyzed the levels of RNAPII at subtelomeres in wild type and *rap1-17* mutant and no difference was observed, suggesting no increase in transcription at these loci (Figure 5b).

Altogether these data indicate a role for Rap1p in post-transcriptional regulation of *subTERRA*.

### 2.6. subTERRA Expression is Cell-Cycle Regulated

**Figure 6:**
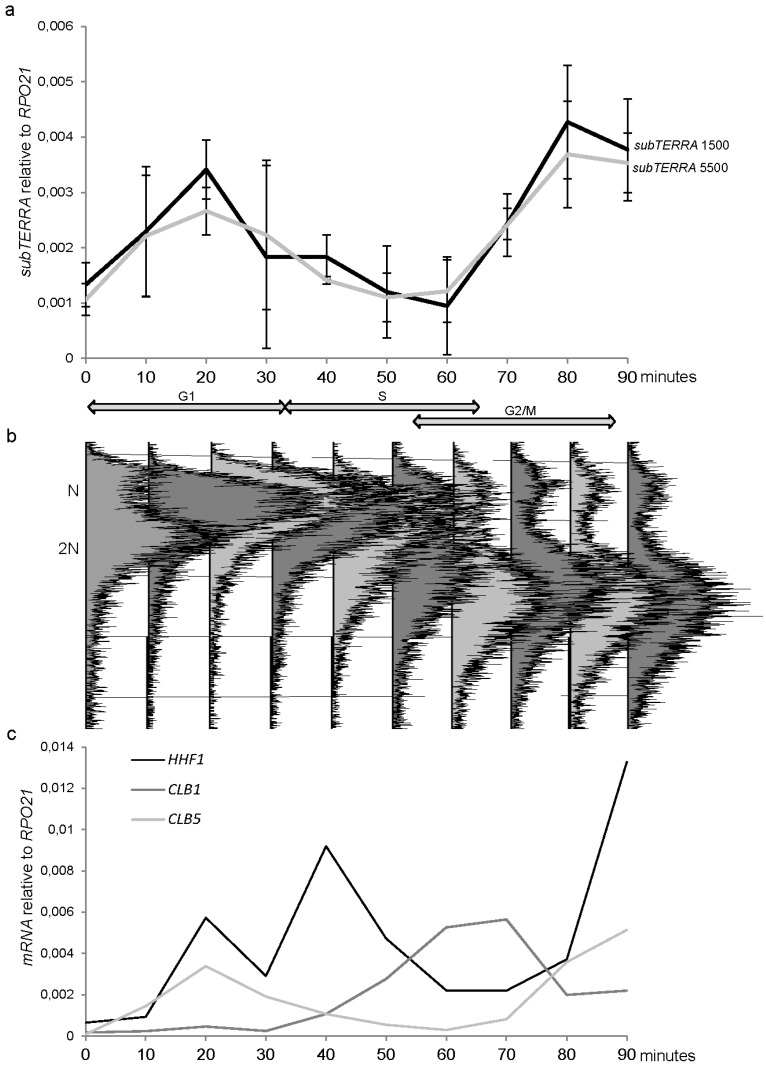
*subTERRA* accumulate in G1/S phase of the cell cycle. (**a**) WT *bar1*∆ cells were grown to exponential phase in YPD medium, diluted and synchronized with *alpha-*factor. Cells were released into the cell cycle by two subsequent washes with YPD with protease. Time course was performed in fresh YPD at 30 °C during 90 minutes with points at each 10 min. Total RNA was extracted and quantity of *subTERRA* was quantified using random primer RT and qPCR with specific primer at 1500 (black line) and 5500 bp (dashed line). Our fold accumulation was observed at time point 20 min corresponding to late G1. Experiments were made twice; (**b**) Cell synchronization was verified by microscopy and, FACS analysis; (**c**) mRNAs, specific for indicated cell cycle phases were amplified using random primers and quantified by qPCR.

To address the role of *subTERRA*, we first aimed to determine whether these RNAs accumulated in wild type cells under certain conditions. As the subtelomeric regions play a role on telomere replication and telomere metabolism, we tested whether *subTERRA* were present at different stages of the cell cycle. WT cells were arrested in G1 in the presence of the mating-type *alpha*-factor. After release of the cells, samples were collected every 10 min and the cell cycle monitored by FACS (Figure 6b). Total RNAs were extracted from each time-point and analyzed by Northern blot with subtelomeric probe and by RT-qPCR (Figure 1a, PCR1 and PCR2). *subTERRA* levels increased progressively up to the maximum (4-fold) in late G1 and decrease afterwards to a minimum as cells came out of the S-phase (Figure 6a). Strand specific analysis is shown at Figure S2c.

Thus, *subTERRA* accumulate in cells before they enter into S phase or before replication starts. Elevated *subTERRA* levels or their transcription could be necessary for establishment of opened replication-prone structures at subtelomeric regions or formation of RNP-molecules specific for S-phase and required for telomere replication and elongation by telomerase. To achieve different levels of *subTERRA* during cell cycle *subTERRA* expression could be regulated through adjustments in transcription and/or degradation rates guided by formation of different RNP complexes.

### 2.7. subTERRA-CUTs are Important for the Telomeric Silencing

**Figure 7:**
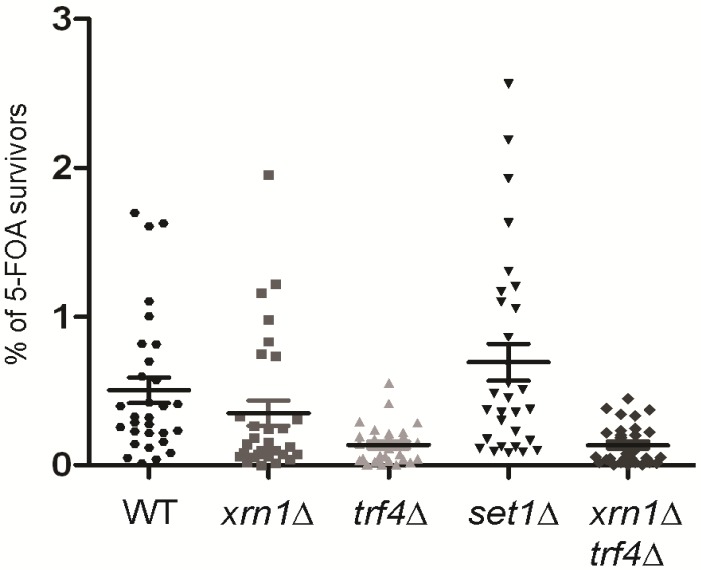
Trf4p-dependent accumulation of *subTERRA*-CUTs enhances telomere position effect. To measure TPE, *XRN1* and *TRF4* genes were deleted in WT strain bearing *telIXL-URA3* reporter (position 7 about 4 kb from telomere end; [61]). Overnight, exponentially grown cells were plated either onto CSM or CSM + 5-FOA plates. Clones were counted after three days of growth at 30 °C. Percent of 5-FOA survivors were normalized to CSM clones, experiments were repeated three times. WT/*trf4*∆ strain *p*-value = 0.00015; WT/*xrn1*∆ *p*-value = 0.21112; WT/*xrn1*∆ *trf4*∆ *p*-value = 0.00014; WT/*set1*∆ strain *p*-value = 0.21971 (Student’s *t*-test).

In order to establish a role of *subTERRA* in telomere maintenance, we studied the effect of RNA decay mutations on TPE (silencing of subtelomeric sequences) [10]. We used native reporter systems with a *URA3* reporter gene integrated in non-modified subtelomere Y′ sequences at telomere IXL [61]. The percentage of 5-FOA (5-fluoroorotic acid) survivors, clones efficiently repressing/silencing the *URA3* gene present in subtelomeric regions was measured. *trf4*∆ mutation significantly decreased the percentage of survivors (*p*-value = 0.00015; Figure 7). This means that the lack of Trf4p leads to less-efficient silencing at subtelomeres. A slight, but not significant effect (*p*-value = 0.21112) of decrease in TPE was also observed in the *xrn1*∆ strain but no synergy could be seen for the double *xrn1*∆ *trf4*∆ strain, suggesting that these mutations are epistatic (*p*-value = 0.00014; Figure 7). The possibility that observed phenotypes are biased by nucleotide metabolism and do not reflect silencing efficiency but metabolic changes caused by studied mutation [62] cannot be excluded. We have tried to address this possibility by testing different *trf4*∆ and *xrn1*∆ strains for their sensibility at 5-FOA-containing medium, independently of subtelomeric reporter expression. No defect of growth was observed on standard 5-FOA concentrations (0.2%–0.24%, data not shown).

These results suggest a role of *subTERRA*, and especially *subTERRA*-CUTs, in telomere silencing.

### 2.8. subTERRA-XUTs Accumulation Counteracts Telomeric Clustering 

**Figure 8:**
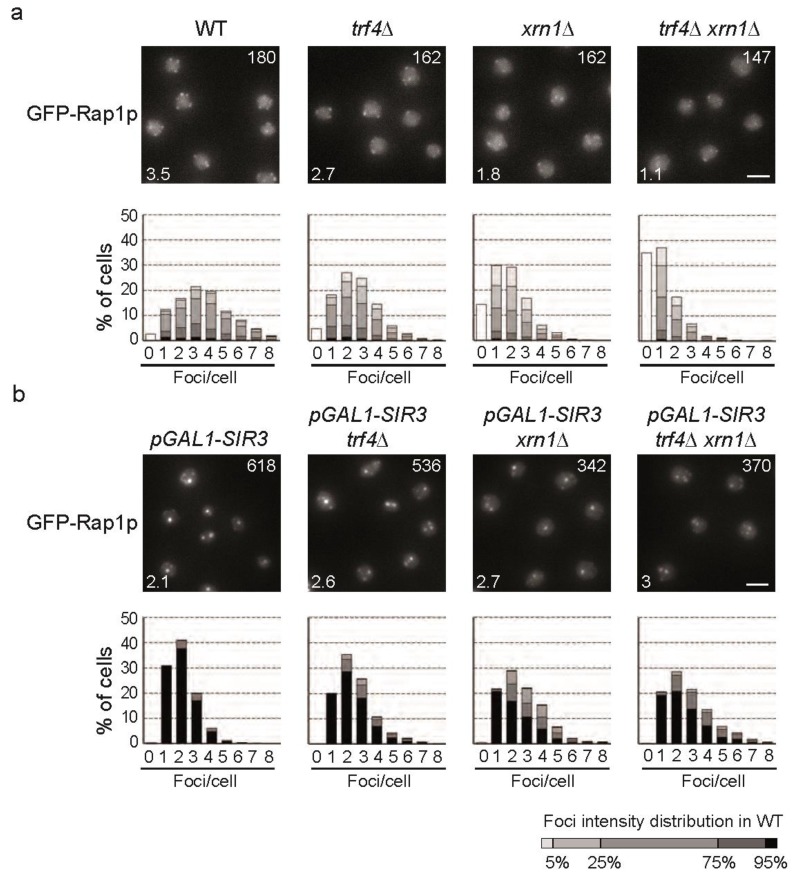
The *xrn1*∆ strain, accumulating specifically *subTERRA*-XUTs is defective for telomeric clustering. (**a**) GFP-Rap1p foci grouping in RNA decay mutants. Representative fluorescent images of the telomere-associated protein Rap1 tagged with GFP of the WT, *xrn1*∆, *trf4*∆ and *xrn1*∆ *trf4*∆ *s*trains. Cells were grown in galactose overnight, diluted to OD_600nm_ = 0.2, and imaged at OD_600nm_ = 1. White number in upper right corner = mean intensity of detected foci; white number in bottom left corner = mean number of detected foci. Images were quantified using *Q-foci* application [35]. Gray levels are set to represent the distribution of foci intensity in wild type cells. Bars, 2 μm; (**b**) Hypercluster formation mediated by Sir3p overexpression is less efficient in RNA decay mutants. Experiment performed as in 8a, but strains were grown in galactose to induce *pGAL-SIR3* [35].

To confirm a role of *subTERRA* in telomere metabolism, we asked whether telomere anchoring and clustering was affected in RNA decay mutants. During the cell cycle, senescence and ALT formation, telomere’s anchoring to nucleopores changes [63,64,65]. The current models propose that telomere tethering through Nup84/Slex5/Slex8 is a prerequisite for the final choice of the recombination repair pathway [66]. In addition, telomeres form discrete foci (three to five foci per nucleus in haploids) at the nuclear periphery that can be easily detected by imaging GFP-Rap1 protein associated with telomere repeats [31,32]. We microscopically analyzed whether telomere foci distribution and intensity changed in cells accumulating *subTERRA* using our in-house application for foci quantification, *Q-foci* [35]. GFP-Rap1p foci were monitored in WT, *xrn1*∆*, trf4*∆ and *xrn1*∆ *trf4*∆ strains (Figure 8a). Our results show that telomere clustering is affected in mRNA decay mutants. *trf4*∆ shows a 23% decrease in telomere foci intensity compared to wild type, which reflects a decrease in the number of telomeres per cluster. *xrn1*∆ has a more pronounced defect. Even if the mean intensity of the foci were similar to *trf4*∆, fewer foci were detected (1.8 instead of 3.5 in a WT and *trf4*∆) indicating that more telomeres are invisible (single or pair) [35]. Indeed, in *xrn1*∆ cells, 45% of the nuclei had one focus or no focus compared to only 15% in a wild type. Remarkably, there is a synergistic effect of the double mutant in which the mean intensity of the foci is strongly decreased compared to WT (41% less intense). Also the number of detected foci is extremely low (1.1 foci instead of 3.5 in WT) with 70% of the cells with one focus or no focus at all. Since DNA replication has been shown to interfere with telomere clustering [32], we also carried out the same experiment in G1 and early S phase cells and obtained the same results (Figure S5).

Next, we tested whether Sir3p overexpression (driven by the strong, inducible promoter *Gal1p-SIR3*; Figure 8b) could restore clustering in *trf4*∆, *xrn1*∆ and *xrn1*∆ *trf4*∆ mutants. In a WT strain, Sir3p overexpression leads to increased telomere clustering [35]; 70% of the cells have one or two extremely bright telomere clusters with a mean intensity of 618 compared to 180 in the WT with the endogenous Sir3 promoter. Deletion of *TRF4* leads to a slight decrease of the mean intensity (13%), but still significantly affects the number of clusters observed per nuclei – 45% of the cells with more than two foci, while this fraction represents less than 30% of the nuclei in a wild type. Deletion of *XRN1* strongly impacts telomere clustering upon Sir3p overexpression. Fifty percent of the cells have more than two foci and the mean intensity of the foci is strongly decreased (45%). Contrary to what we observed with endogenous Sir3p level there is no synergistic effect of the combination of *xrn1*∆ and *trf4*∆ mutants upon Sir3p overexpression. This suggests that *subTERRA* accumulation impinges on telomere clustering upstream of Sir3p action.

Recapitulating, *xrn1*∆ strongly affects telomeres clustering while *trf4*∆ has only a weak effect. This suggests that the 5′end RNA decay pathway has a predominant role over the 3′end RNA decay pathway in the maintenance of telomere clustering. We hypothesized that only *subTERRA-*XUTs, transcribed towards the telomeres, would be prone to de-clusterization of telomeres and a decrease of telomere-telomere interactions. To prove this hypothesis we used *subTERRA* overexpression from its chromosomal loci (Figure S6a) to determine whether *subTERRA* plays a direct role in telomere clustering. Upon galactose induction, GFP-Rap1p foci were quantified and no significant effect of this *trans*-expression of antisense *subTERRA* was observed (data not shown). We conclude that despite the possible role of *subTERRA*-XUTs in telomere clustering its action could be limited to *cis* mode of regulation and therefore undetectable in a global manner.

### 2.9. xrn1∆ and trf4∆ Mutations Change Global Expression Profiles but do not Affect Specifically Telomere Homeostasis Genes

**Figure 9:**
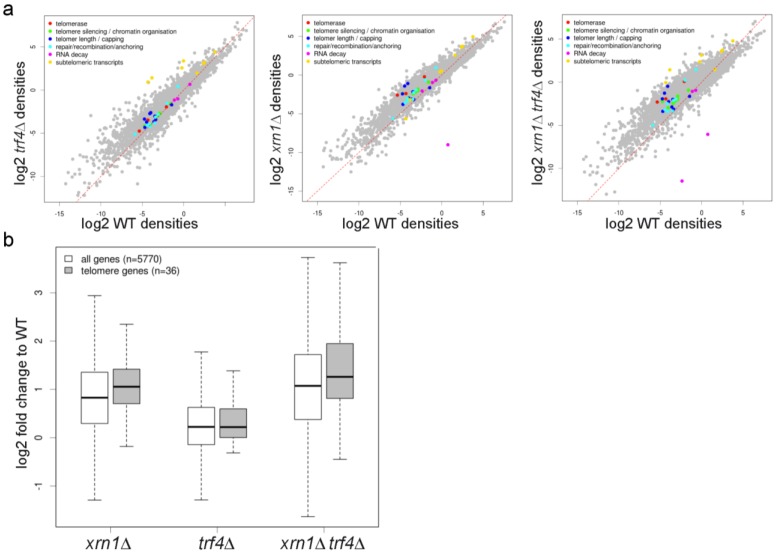
*xrn1*∆ and *trf4*∆ impact the general transcription profile but do not change specifically expression of genes important for telomere maintenance. (**a**) Expression of different classes of genes implicated in telomere maintenance in wild type, *trf4*∆, *xrn1*∆ and *xrn1*∆ *trf4*∆ cells shown as scatter plot of tag density for selected genes in wild type strain *vs.* *xrn1*∆, *trf4*∆ and *xrn1*∆ *trf4*∆ strain. Results are presented as log_2_ of density, expressed in tag per nucleotide; (**b**) Global expression of all protein coding and telomere-maintenance in mutants deleted for RNA decay factors (group of 36 genes). Box plot representation of expression fold change for all protein-coding (white) and telomeric (gray) genes in wild type, *xrn1*∆ and *trf4*∆ mutants. The black line within the box corresponds to the median value; the top and bottom lines correspond to the upper quartile and lower quartile, respectively.

*subTERRA* accumulate in mutants lacking Xrn1p, a major cytoplasmic exonuclease, which in complex with Dcp1/2p, Lsm1-7p, represents a major mRNA degradation apparatus [67] devoid of Trf4p, a poly-A polymerase modulating the activity of nuclear exosomes [68]. Since the lack of Xrn1p provokes an increase of coding transcripts [47] therefore the *xrn1*∆ mutant as well as double mutant, lacking Xrn1 and Trf4 proteins, affects the global profiles of gene expression and consequently the genes dedicated to telomere metabolism. RNA-seq experiments (described in paragraph 2.2) confirmed the effect of *xrn1*∆ mutation on transcriptome (2.0-fold increase of coding transcripts). In the case of *trf4*∆ mutation ORF transcripts increased up to 1.5-fold and the double mutant showed 2.7-fold accumulation of mRNAs (Figure 9a). This analysis suggests that cytoplasmic and nuclear degradation machineries act independently on the majority of cellular transcripts since double mutants showed an increased level of all types of analyzed RNAs: ORF, CUTs, SUTs, XUTs, sn/snoRNAs, tRNAs (Figure 9a). Expression of genes involved in telomere metabolism (group of 36 genes, see Material and Methods) was affected to the same extent (less than 5% in the test of Kolmogorov-Smirnov for goodness of fit, indicated that telomeric genes follow the same changes as that of all ORFs; *xrn1*∆ *p*-value = 0.06814; *trf4*∆ *p*-value = 0.2634; *xrn1*∆ *trf4*∆ *p*-value = 0.1792; Figure 9a,b). Functions of these proteins can be roughly categorized into those affecting telomerase activity (e.g., *EST1, EST2, EST3, TLC1, KU70*/*KU80, PIF1*, and the MRX complex, reviewed in [69]); those that affect stability of telomerase RNA – *TLC1* (e.g., *NMD1/2/3*, *MTR10*) [70,71]; and those that play a role in the regulation of telomeric heterochromatin, replication, or end protection (e.g., *SIR2/3/4*, *CDC13*, *STN1*, *TEN1*, *RAP1*, *RIF1*, *RIF2*, *MEC1*, and *TEL1*) [69].

*trf4*∆ and *xrn1*∆ mutants show important differences in telomere-associated phenotypes, which could not simply be explained by observed changes in the transcriptome. For example as previously described, we confirmed that *xrn1*∆ [72] and *trf4*∆ [73] strains have telomere extremities slightly shorter than a wild type, while the combination of the two mutations leads to a significant reduction of telomere length similarly to the *set1*∆ strain, known to affect telomere size [74]; (Figure S7). Cells deleted for *yKU70/80*, *TEL1*, *MRE11*, *RAD50*, *XRS2*, *EST1/2/3* have very short telomeres [72] and in the *xrn1*∆ strain, these mRNAs accumulate more or less significantly (1.3/2, 2.5, 2, 2, 1.3. and 3.8/6.8/3.6-fold, respectively) but their telomeres are short. On the other hand the *trf4*∆ strain, with telomeres even shorter than *xrn1*∆, Figure S4) accumulates the same transcripts to a lower extent or not at all; *yKU70/80 – 0.8/1.1*, *TEL1 – 1.4*, *MRE11 – 1.2*, *RAD50 – 1*, *XRS2 – 1.1*, *EST1/2/3* – 1.8/1.5/1.1-fold, suggesting that the variation of these genes cannot explain fully the phenotypes of the strains.

There are at least two possibilities explaining these observations (*i*) subtle equilibrium of steady state levels of mRNAs encoding for telomerase and telomere-associated proteins would change the stoichiometry of telomerase complex subunits, telomere capping structures or heterochromatin organizing complexes. In this case, simple correlation of RNA levels could not explain observed changes of phenotype; (*ii*) changes in mRNA levels would not be translated into protein level changes as previously shown on other examples of RNA-decay phenotypes [49,68,75]. In this case *subTERRA* could participate for the changes in telomere length. To address this hypothesis, we over-expressed individually different *subTERRA* species using an inducible promoter (*GAL1-10;* Figure S6a). Our results show no difference with the non-induced conditions (Figure S6b). Several explanations could account for this output. First a transient expression might be too short to control telomere erosion; second the formation of double-stranded *subTERRA* RNA would be required to control telomere elongation or finally *subTERRA* would not be able to act in *trans*.

**Figure 10:**
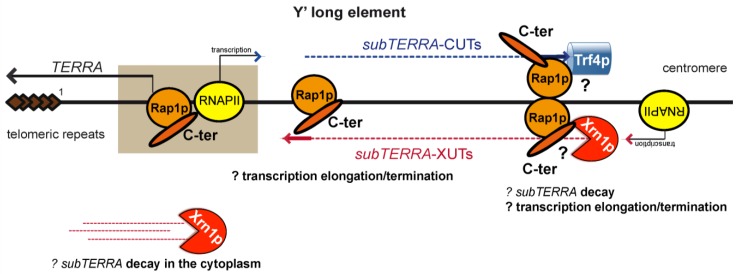
View of *subTERRA* regulation. We propose *subTERRA* as a novel factor impacting structure of chromosome end. In agreement with the platform properties the Rap1-C-terminal domain (Rap1p orange circle and C-terminal domain orange oval) appears as a hub recruiting degradation machineries *i.e.*, Trf4p and Xrn1p or other, unidentified factors. Described negative regulators of *subTERRA* are proposed to control *subTERRA* decay, transcription elongation or termination. *subTERRA*-dependent recruitment of these proteins to subtelomeric region changes its structure impacting on telomere dynamics (telomere-end looping, TPE, clustering, telomere length). XUTs, transcribed towards centromere are red and CUTs transcribed towards the telomere-end are blue. In light brown we have underlined the region of *TERRA/subTERRA* discontinuity, containing potentially promoters and terminators of different lncRNAs.

## 3. Discussion

With this work, we show that several long, unstable RNAs are produced within the budding yeast subtelomeric Y′ region. We defined these lncRNAs as *subTERRA* since, contrary to *TERRA* they do not cover the terminal telomeric repeats. *subTERRA* are transcribed in both transcriptional senses which gives rise to two sets of RNAs: *subTERRA*-CUTs, sensitive to nuclear degradation are transcribed towards centromere, and *subTERRA*-XUTs are preferentially degraded in the cytoplasm by Xrn1p.

### 3.1. Characterization and Subcellular Localization of subTERRA

*subTERRA* localize mainly to the nucleus in *xrn1*∆ *trf4*∆ mutant, which indicates that their function is associated with their transcription site. The role of subtelomeric regions is not clear. Y′ elements may contribute to different aspects of telomere maintenance, but this potential role is only unmasked when some aspect(s) of telomere maintenance are compromised; like during aging, particular growth/adaptation conditions or cell cycle phase. If the biological role of *subTERRA* were telomere maintenance (loop folding, capping, nuclear membrane binding, recombination), the nuclear localization would be expected. It would be of high interest to address the localization of the different families of *subTERRA* and their co-localization with different nuclear components upon physiological conditions. Recent experiments addressing this question for *TERRA* suggested that yeast *TERRA* (*yTERRA*) transcribed from a critically short telomere functions as a scaffold for the formation of telomerase clusters (T-Recs; *telomerase* *recruitment* *clusters*), which are subsequently recruited to telomeres [76]. Live-cell imaging analyses of *TLC1* RNA revealed that telomerase nucleates emerge in G1/S phase at the nuclear periphery, generating T-Recs, which subsequently co-localize with telomeres [77]. These data bring out *yTERRA* as a positive regulator of telomerase-dependent telomere elongation predominantly in *cis,* ensuring that only short telomeres become substrates for telomerase-mediated elongation.

We show here that *subTERRA* localize to the nucleus; detected signals were weak and dispersed in the WT cell and aggregating in the nucleus as punctuated structures in *xrn1*∆ *trf4*∆ mutant. This is comparable with *TERRA* signals described by Cusanelli and colleagues [76]. Only a small population of WT yeast cells expressed *TERRA*. More specifically, Y′-*TERRA* (transcribed from all the Y′-element containing telomeres) was mostly detected as double or multiple foci in about 30% of the cells [76]. In the case of *subTERRA* we estimated that in the *xrn1*∆ *trf4*∆ mutant 25 to 40% of cells accumulated the *subTERRA* signal in the nucleus and we cannot exclude that there is a low percentage (10%) of WT cells with nuclear signal. Cell cycle studies as well as improved specificity of detection of different *subTERRA* families would be necessary to address their role.

### 3.2. subTERRA Expression and Mode of Action

*subTERRA* expression is cell-cycle regulated and peaks at late G1/early S phase, mainly as a result of its increased stabilization, then continuously decreases as cells progress through S phase to finally be re-established after mitosis, similarly to human *TERRA* [78]. ChIP-RNAPII on synchronized cultures showed no increase in occupancy indicating at least that RNAPII initiation is not activated (data not shown). In yeast, telomerase is constitutively expressed but its activity is predominantly detected during the late S/G2 phases, coupled with the replication of telomeres [79], which anti-correlates with *subTERRA* maximum level. This indicates that the role of *subTERRA* and its transcription would be important before replication and one can imagine that presence of subtelomeric transcription and *subTERRA* would promote telomere conformation(s) excluding telomerase accessibility. This can be achieved by changing telomere:subtelomere structure by formation of loops stabilized by protein:protein and RNA:DNA interactions. Later on, during late-S phase the bulk of telomere sequences are replicated [80] and telomeres, anchored to the nuclear envelope during interphase/G1, are released from perinuclear foci by progressing replication and yKu repression [81]. Moreover, it is only during late S-phase that telomerase accumulates, preferentially on short telomere/s, and forms a processive structure called T-Rec (telomerase recruitment cluster; [9]). In this window of the cell cycle *subTERRA* levels are at the lowest and telomere structure is changed permitting chromosome segregation. The gathered observations indicate that *subTERRA* action is rather in *cis*, implicating changes in telomere structure. Several arguments allow reinforcing this hypothesis; two telomeric phenotypes potentially affected by *subTERRA* *i.e.*, TPE and telomere clustering implicate *cis* action of *subTERRA.* RNAPII transcription, within heterochromatin regions, is repressed by the Sir2 deacetylase, and even though these regions are not theoretically accessible for transcriptional machinery, RNAPII is present and low transcription is taking place [50]. In wild type cells *subTERRA*-CUTs are probably transcribed at a low/basic level and rapidly degraded in the nucleus by the Trf4p-dependent pathway, which leads to variegation. In this model TPE would be controlled in *cis* by progressing transcription impacted by Trf4p itself or by degradation efficiency of its products— *subTERRA*. *subTERRA*, acting in *cis,* could interfere with transcription efficiency by recruiting protein complexes/regulators of RNAPII or chromatin modifiers immediately after RNAPII passage. We propose that *subTERRA* are produced to facilitate the establishment of specific heterochromatic states of subtelomeric chromatin.

In yeast, telomerase is constitutively expressed but upon its deletion/inactivation, critically short telomeres induce a senescence-like phenotype [82,83,84]. As suggested for *yTERRA,* for which increased levels are prevalent in surviving yeast and could promote survivor establishment and telomere lengthening [85,86], *subTERRA* levels also increases in ALT survivors, but not in pre-senescent, telomerase negative cells. Y’ element, amplified in ALT1 cells, is transcribed, which give rise to increased *subTERRA* level and at the same time processing transcription opens newly created chromosome ends. The use of transcription-inducible telomeres (tiTELs) in mammalian cells [87] and in yeast [78,88] enabled intense transcription of telomeric tracts and increase in the levels of *TERRA*. These data showed that intense transcription resulted in a shortening of the transcribed telomere in *cis* and increased rates of telomere recombination [89]. Interestingly, the shortening of a yeast tiTEL was reduced in telomerase positive cells compared to telomerase negative controls [88]. One can imagine that *subTERRA* further stabilizes the opened chromatin state by interacting with DNA forming RNA:DNA hybrids and at the same time enabling recombination. As RNaseH has been shown to impact *TERRA*-telomeric DNA hybrids and telomere maintenance in ALT tumor cells [90] and telomere shortening and *TERRA* accumulation in yeast [40], it will be of interest to check the impact of RNaseH on *subTERRA* levels in senescent cells. A recent report provides evidence that homologous recombination events between sister chromatids can counteract the accumulation of ssDNA in the subtelomeric region of short yeast telomeres and delay checkpoint activation [91]. This suggests the role of subtelomeres in telomere maintenance during senescence.

### 3.3. Role of Rap1p in subTERRA Expression

In this study we show that the Rap1p C-terminal domain is a destabilizing factor for *subTERRA* lncRNAs. This effect is independent of SIR proteins and is not related with elongated telomeres (no changes of *subTERRA* in *rif1/2*∆ mutants presenting very long telomeres). Rap1p binds specific telomeric repeats but also at least four other types of sequences dispersed in the genome. The efficiency of Rap1p binding and its turnover on these loci varies, which in consequence determine the mode of action of Rap1p [92]. Dispersed and degenerated TG repeats are present all over subtelomeres and were proposed to serve as a platform for creating telomeric loops. This possibility is confirmed by the impact of Rap1p on *subTERRA*. Since the *rap1* mutant stabilizes *subTERRA,* Rap1p acts here as a negative regulator *i.e.*, it promotes *subTERRA* degradation. One can imagine that Rap1p would be important for the formation of secondary structures involving subtelomeric DNA and *subTERRA;* during G1 phase *subTERRA* levels are high and telomeres in closed conformation, Rap1p-dependent *subTERRA* degradation is slow. It was recently shown [93] that a *cis* element, comprising two Rap1p-binding sites, and Rap1p itself, are necessary and sufficient to induce enhanced decay of the reporter mRNA and that Rap1p stimulates both the synthesis and the decay of a specific population of endogenous mRNAs. The impact of Rap1p on *subTERRA* could depend on its dynamics in the cell during cell cycle and on its affinity to the *subTERRA*. It is not excluded that Rap1p binds directly *subTERRA* and very probably binds *subTERRA* promoters. In agreement with recent discoveries, Rap1p could affect the export of *subTERRA* RNAs, which in turn would regulate its decay in the cytoplasm. Further study is needed to determine whether Rap1p could coordinate rates of synthesis and decay of RNAs, and its impact on *subTERRA* and telomere metabolism.

## 4. Conclusions

We propose that *subTERRA* play a role(s) in modulating some aspects of the telomere metabolism to enable sufficient fluidity and reversibility of the telomere structure. An attractive hypothesis is that *subTERRAs* modulate telomere anchoring, being an early actor in the choice of telomeric and subtelomeric recombination events after telomere erosion. The scaffold-like function of *subTERRA* may be similar to what has been proposed for a growing number of lncRNAs. *subTERRA*, as *TERRA*, could play a role in the recruitment of protein complexes, and the formation of clusters that further organize maintenance processes such as recombination, transcription or capping.

## 5. Material and Methods

### 5.1. Yeast Strains and Plasmids

The strains used in this study are described in Table S1. The experiments were mostly performed in the derivatives of BY4741 (S288C, Euroscarf, Frankfurt, Germany) or W303 [94] backgrounds. Gene deletions and insertions of alternative promoters were performed by PCR-based gene targeting [95,96]. Transformants were verified by PCR using upstream and downstream external primers. For Xrn1p and Trf4p catalytic mutant experiments, the *xrn1*∆ and *trf4*∆ strain was transformed with a plasmid carrying wild type *XRN1* (PAM27, pAJ52)/*TRF4* (PAM177) gene or *xrn1-E176G* (PAM143, pAJ53)/*trf4-236* (PAM178) mutated allele (a gift from A. Johnson and M. Christman; [55], respectively) or with empty vectors. Transformed cells were grown in CSM (Complete Synthetic Media; MP Biomedicals France) selective media with glucose at 30 °C, heat shock was performed at 37 °C.

### 5.2. Media and Culture Conditions

Growth media and plates were prepared with standard methods using YPD or CSM media, supplemented as indicated and containing 2% glucose. Standard growth conditions were employed (30 °C), heat shock was performed at 37 °C. For galactose induction in rich medium, cells were pre-cultured in YPD and switched to YPGal medium (2% galactose) for induction of the *GAL1* promoter. Telomeric silencing assays were carried out as follows: yeast cells were grown on appropriate selective media (CSM) and were dropped on CSM plates containing 0.1% of 5-FOA (Zymo Research Europe, Freiburg, Germany). Cell cycle experiments were performed in YPD media using *bar1*∆ strain (Euroscarf) grown at 30 °C. 5 μM *alpha*-factor mating pheromone (Zymo Research Europe,) was added to exponentially grown cultures of OD_600_ = 0.2–0.4. After 90 min of synchronization (verified under microscope with a shmoo formation efficiency of 90%–100% confirmed in G1-arrested cells) cells were spun down and washed twice with YPD. Fifty µg/mL of pronase (Roche Diagnostics Indianapolis, IN, USA; pronase did not affect cell growth) was added after the last wash and cells entered the cell cycle. Cells were collected every 10 min and separated into three parts for ChIP analysis (formaldehyde fixation), RNA extraction (hot phenol extraction) and fixed in 70% ethanol at 4 °C for FACS analysis. For FACS analysis DNA was labeled with propidium iodide (PI), cells were RNase treated and sonicated.

### 5.3. RNA Extraction, poly(A)^+^ RNA Purification and Northern Blotting

Total RNA was extracted using the hot phenol extraction method from 10- to 50-mL of cultures at OD_600_ = 0.4–0.8, followed by contaminant DNA removal using RNase-free DNaseI concentration was measured on a Nanodrop spectrophotometer. In the specificity control experiments RNA was treated with RNaseA. poly(A)^+^ RNAs were purified on oligo(dT) Dynabeads (Invitrogen, Life Technologies, Cergy Pontoise, France). Unless otherwise stated, 10 μg of total or poly(A)^+^ RNAs were loaded on a denaturing 1% agarose gel and transferred to nitrocellulose membranes (Amersham Hybond-N+, GE Healthcare Life Sciences, Velizy-Villacoublay, France). Membranes were UV-cross-linked and hybridized overnight with ^32^P-labeled DNA probes (single-stranded at 42 °C or double-stranded at 65 °C) in PerfectHyb™ Plus Hybridization Buffer (Sigma, Lyon, France). Blots were washed twice with 0.1 × SSC and 1% SDS for 20 min at 37 °C or 65 °C, respectively. Probes were obtained by random-primed labeling (Prime-It Random Primer Labeling Kit, Stratagene, Agilent Technologies France, Les Ulis, France) of specific DNA fragments generated by PCR or kinase labeling of locus-specific oligonucleotides. PCR primers are available upon request. *18S-rRNA*, *ACT1, RPO21* and *scR1* RNAs were used as loading controls. Northern blot signals were quantified using Quantity One software (Biorad).

### 5.4. Reverse Transcription and qPCR

For RT-PCR reaction, 100–500 ng of total RNA was subjected to reverse transcription (SuperScript II Reverse Transcriptase, Invitrogen) with locus-specific or random primers for 45 min at 42 °C. Quantitative PCR was performed with the LightCycler 480 (Roche) using LightCycler® 480 SYBR Green I Master (Roche). PCR were normalized with *18S-rRNA*, *ACT1, RPO21* and *scR1* signals. Error bars correspond to standard deviation over three independent cultures.

### 5.5. RNA-seq

#### 5.5.1. Preparation of Libraries

Libraries were prepared as described before [47]. Yeast cells were grown to OD_600_ = 0.4–0.8 in YPD medium. Total RNA was extracted using the hot phenol extraction procedure. Ribosomal RNAs were depleted from 10 μg of total RNA using the RiboMinus™ Eukaryote Kit for RNA-Seq (Invitrogen). Quality of total and rRNA-depleted RNA was checked by Northern blot and with a RNA Pico 6000 chip in an Agilent 2100 Bioanalyzer. RNase III fragmentation was performed according to the SOLiD^®^ Total RNA-Seq Kit protocol (Applied Biosystems, Life Technologies, Cergy Pontoise, France), starting from 750 ng of rRNA-depleted RNA. Fragmented RNA were cleaned up using the RiboMinus™ Concentration Module (Invitrogen) and eluted in 20 μL of nuclease-free water. Running a sample of fragmented RNA on a RNA Pico 6000 chip in an Agilent 2100 Bioanalyzer checked RNA fragmentation. RNA-Seq libraries were constructed using the SOLiD^®^ Total RNA-Seq Kit (Applied Biosystems) according manufacturer’s instruction, starting from 60 ng of fragmented RNA.

#### 5.5.2. Data Mapping

Fifty nucleotide-long sequence reads (performed on an ABI SOLiD^®^ v4 sequencer) were identified using the standard SOLiD base-calling software and then aligned to the reference genome (*Saccharomyces cerevisiae* S288c retrieved from the Saccharomyces Genome Database (SGD) [97] using Bowtie (v0.12.7) software allowing up to three mismatches.

#### 5.5.3. Transcriptome Analysis and Normalization

Read densities were computed for each transcript in each sample and in all cases only tags mapping to unique positions were considered. Each repeated sequence was mapped separately on its own sequence. Tag densities were normalized on the total reads mapped on all ORFs. For 4 strains we disposed 4 sets of runs of different sequencing depth; WT and *xrn1*∆ 55.175.997 and 37.582.695 reads, and for *trf4*∆ and *xrn1*∆ *trf4*∆ 12.038.301 and 16.249.300 reads. In order to compare these sets, individual weight, depending on total read number of this set, has been attributed to each subtelomeric read. It is important to note that repetitive sequences are represented as one reference genome and not reflecting each of the repeats; our mapping cannot discriminate between all the repeats. On this artificial Y′ genome we performed segmentation process (ZINAR script; [98]). Briefly, it is based on the number of tags and a sliding window that detects changes in density of tags. Overlapping reads are summed beyond 10 reads; one segment is formed of more than 150 nt. Based on RNA-seq profiles we have manually identified four different RNAs. Sequence data are publicly available at Gene Expression Omnibus (GEO) accession number: WT – 1sra/SRX046705 and 2sra/SRX046709; *xrn1*∆ – 1sra/SRX046706, 2sra/SRX046710; *trf4*∆ and *xrn1*∆ *trf4*∆ – GSE67585 at http://vm-gb.curie.fr/marta/XUT_trf4/. Differential expression analysis was performed using DESeq [99]. Telomeric genes category consist of *EST1, CDC13, RAT1, SIR2, EST2, STN1, NMD1, SIR3, EST3, TEN1, NMD2, SIR4, TLC1, MEC1, NMD3, SAS2, RIF1, MTR10, SAS4, RIF2, TEL1, SAS5, RAP1, PIF1, SET1, MRE11, DOT1, RAD50, RAD6, XRS2, RAD52, RAD51, YKU70, YKU80, ESC1.*

### 5.6. Chromatin ImmunoPrecipitation (ChIP)

ChIPs were performed essentially as described previously [44,100]. Yeast strains were grown to OD_600_ = 0.4–0.8 in YPD or CSM media at 30 °C, and cross-linked for 10 min by the addition of formaldehyde to a final concentration of 1.2%. Adding glycine quenched the cross-linking reaction. Chromatin was sonicated to obtain 400–500 nucleotides DNA fragment and 100 μg of sonicated chromatin (protein content) was immunoprecipitated for 3h at 21 °C on Pan mouse Dynabeads (Invitrogen) coated with specific antibody against the carboxy-terminal domain of Rpb1p (8WG16, Millipore, Fontenay sous Bois, France). Immunoprecipitated DNA was quantified by real-time PCR using the LightCycler 480 (Roche) with primer pairs (sequences available upon request). Signals are expressed as percentage of input DNA relatively to *RPO21* (coordinate: chromosome 4: 210562 to 205361). Error bars correspond to standard deviations of three independent cultures.

### 5.7. Fluorescence In Situ Hybridization (RNA-FISH)

FISH was performed according to [35], with a few modifications. The probe was obtained by PCR on a plasmid containing 4.8 kb of Y′ element (pEL42H10; [101]) with couple of primers: am151-GAAGAATTGGCCTGCTCTTG/am152-CCGTAAGCTCGTCAATTATT. The PCR purification was followed by a nick translation labeling reaction using the Nick Translation kit from Vysis (Abbott Molecular, Inc., Rungis, France). The fluorophore used in the reaction was SpectrumRed (Abbott Molecular, Inc.). The probe was denatured for 5 min at 98 °C, purified by ethanol precipitation, and re-suspended in the hybridization mix (50% formamide, 10% dextran sulfate, and 2 × SSC). Cells were grown overnight to mid–logarithmic phase (around 1 OD corresponding to 1 × 10^7^ cells/mL) in 30 mL YPD (30 OD) and harvested at 1200 × *g* for 5 min at RT. Cells were re-suspended in 25 mL of 4% paraformaldehyde for 20 min at RT, washed twice with 20 mL H_2_0, and re-suspended in 2 mL of 0.1 M EDTA-KOH, pH 8.0, and 10 mM DTT for 10 min at 30 °C with gentle agitation. Cells were then collected at 800 *g* at RT, and the pellet was carefully re-suspended in 2 ml YPD and 1.2 M sorbitol. Next, cells were spheroplasted at 30 °C with Zymolyase (8–16 µL Zymolyase 100T at 5 mg/mL to 1 mL YPD-sorbitol cell suspension). Spheroplasting was stopped by the addition of 40 mL YPD and 1.2 M sorbitol. Cells were washed twice in YPD and 1.2 M sorbitol, and the pellet was re-suspended in 1 mL YPD. Cells were dropped on diagnostic microscope slides and superficially air dried for 2 min. The slides were put in methanol at -20 °C for 6 min, transferred to acetone at -20 °C for 30 s, and air-dried for 3 min. The cells were washed with 4 × SSC for 10 min at RT and rinsed twice with 4 × SSC and dehydrated in ethanol 70, 80, 90, and 100%, consecutively at -20 °C for 1 min in each bath. Slides were air dried, and a solution of 2 × SSC and 70% formamide was added for 5 min at 72 °C. After a second step of dehydration, the denatured probe was added to the slides for 10 min at 72 °C followed by incubation for 24 h at 37 °C in a humid chamber. The slides were then washed quickly in 0.05 × SSC and subsequently incubated in 0.05 × SSC at 37 °C for about 60 min and washed with 1 × PBS with DAPI for 10 min at RT. Slides were then washed three times with water and air-dried. Fifteen µL/spot of anti-fading compound in glycerol, pH 7.5 (DABCO) was added before imaging. Before the dehydration step control slides were treated with 20 µg/mL RNase in 4× SSC, 0.1% Tween for a few hours, washed with water and dehydrated. Images were acquired with a wide-field microscope the same day, using identical acquisition parameters on cells grown in the same culture conditions.

### 5.8. Sub-Cellular Fractionation

Hundred fifty OD (500 ml of OD_600_ = 0.35) of cells were collected by centrifugation and washed twice with cold water. Cells were re-suspended in 50 ml of Z buffer (2 mM MgCl_2_; 10 mM NaCitrate pH 7.5; 120 g/L mannitol; 9 mM *beta*-mercaptoethanol) and incubated 30 min at 37 °C and 15 μg of Zymolase 20T was added and cells were spheroplasted for 10–30 min, depending on efficiency of digestion. Spheroplasts were chilled on ice for 10 min and pelleted at 4 °C than washed twice with cold Z buffer. Spheroplasts were re-suspended in 1 mL of cold Z buffer + 0.1% Triton X-100 and samples were broken 20 times using a Dounce homogenizer (15 mL) on ice. 0.7 mL of cold PS1 buffer (Z buffer + 0.6 M sucrose) was added and 100 μL aliquots were taken for total protein extract (TOT). Cell lysate was layered on top of 4 mL of PS2 buffer (Z buffer + 0.45 M sucrose) and centrifuged for 10′ (with 2′ brake and acceleration time included) at 4000 rpm at 4 °C. 500 μL of upper supernatant = CYT1 and subsequent 500 μL = CYT2 were taken and the rest of the supernatant discarded. The nuclear pellet, about 100 μL, is the nuclear fraction = NUC. All buffers contained AEBSF (4-2-Aminoethyl) benzenesulfonylfluoride hydrochloride; Sigma) and RNasin (Promega, Charbonnieres, France). RNA was extracted from total extract and subcellular fractions using the hot phenol method and analyzed by Northern blot using specific probes.

### 5.9. Telomeric Clustering

Yeast cells were grown in synthetic medium (yeast nitrogen base; MP Biomedicals) supplemented with 2% glucose or galactose (wt/Vol) and the appropriate supplement mixture (complete or lacking a nutrient; MP Biomedicals). Liquid synthetic media were enriched for complete synthetic medium (2× complete synthetic medium as final concentration [102]). All the strains were grown at 30 °C. The live-cell images were acquired using a wide-field microscopy system based on an inverted microscope (TE2000; Nikon, Champigny sur Marne, France) equipped with a 100Å~/1.4 NA oil immersion objective, a charge-coupled device (CCD) camera (Coolsnap HQ2; Photometrics, Tucson, AZ USA), and a xenon arc lamp for fluorescence (Lambda LS; Sutter Instrument Co., Novato, CA USA), a collimated white light-emitting diode for the transmission, and a UV filter on the two illumination paths (LP 400 and GG400; Nikon). Green fluorescent images were acquired with a GFP filter block (excitation: band pass (BP), 465–500 nm and dichroic, 506 nm; emission: BP, 516–556 nm; Semrock, Rochester, New York, USA). All fluorescent images are a z projection of z-stack images, acquired with an axial (z) step of 200 nm. Before quantification, deconvolution was made using the Meinel algorithm in Metamorph (eight iterations; Σ = 0.8; frequency 3; MDS Analytical Technologies, Sunnyvale, CA, USA). Analyses have been performed using a home-made Matlab (MathWorks, Natick, MA, USA) application (*Q-foci*) [35]. For manual counting (Figure S5) cells were grown to exponential phase (OD_600_ = 0.8) in YPD at 30 °C. 3D-pictures were acquired as described above and Maximum Intensity z-projection (ImageJ) was applied to manually count GFP-Rap1p foci. Z-series were taken for at least 100 not-budded/small-budded cells.

### 5.10. Telomere Length Analysis

Telomere length was analyzed as described in [103]. Total DNA was isolated from cells grown overnight in YPD (OD_600_ = 2.0). 50–100 µg DNA was digested with *Xho*I (NEB) overnight at 37 °C and run on 25-cm-long 0.8% agarose gels for 18 h at 100V. Restriction-digested fragments, containing *S. cerevisiae* telomeric repeats were loaded with digested DNA as a size control. Southern blotting was performed using Amersham Hybond™-XL membranes (GE Healthcare), and probed with radiolabeled (dCTP, α^32^P, Exo (-) Klenow Polymerase from Stratagene) telomeric probe containing TG_1–3_ repeats. Hybridizations were done in PerfectHyb™ Plus Hybridization Buffer (Sigma) at 65 °C. The average telomeric length was estimated for each lane by calculating the distance from the peak of signal intensity of the telomere band against the position of the added internal size standard (Quantity One 4.6.5 Basic, Biorad).

## Supplementary Files

Supplementary File 1

Supplementary File 2
