# Supplementary material for: Expression of Subtelomeric lncRNAs Links Telomeres Dynamics to RNA Decay in S. cerevisiae
Source: Noncoding RNA. 2015 Jul 3;1(2):94–126. doi: 10.3390/ncrna1020094 (PMC5932542; doi:10.3390/ncrna1020094)
Supplement: Supplementary File 1 [file ncrna-01-00094-s001.pdf]

Supplementary Materials

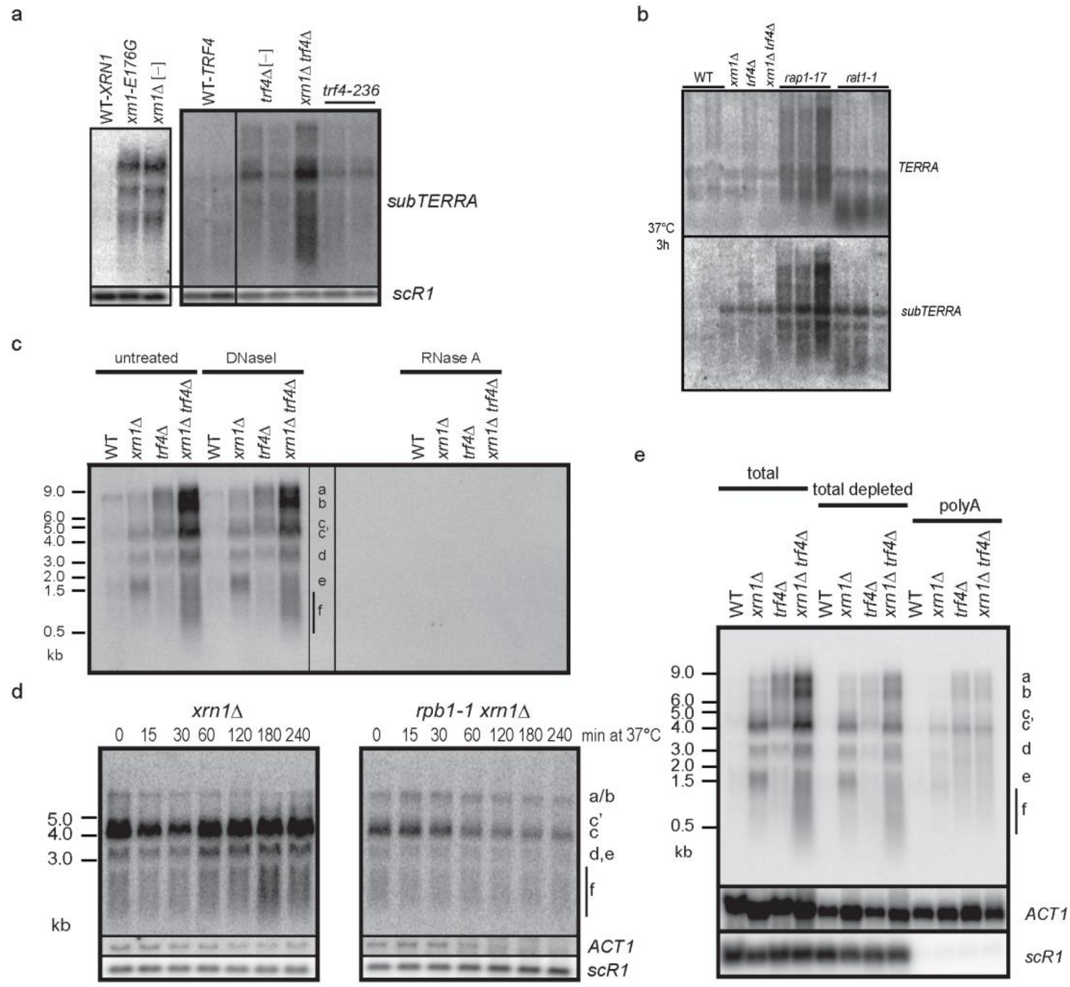

Figure S1. ***subTERRA*** **are lncRNA transcribed by RNAPII, partially polyadenylated and distinct from** ***TERRA*****.** (**a**) *xrn1*Δ (W303) strain was transformed with *LEU2*, centromeric plasmid carrying WT-*XRN1*, *xrn1-E176G* [104] or empty plasmid (pRS315; [28] for *xrn1*Δ [-]; *trf4*Δ (W303) was transformed with *HIS3*, centromeric WT-*TRF4*, *trf4-236* mutant allele [55] and with empty vector pRS413 [105] for *trf4*Δ [-]. RNA was extracted from exponentially grown cultures (CSM-leu and CSM-his, respectively). Double mutant *xrn1*Δ *trf4*Δ was grown in CSM. Northern blot with subtelomeric probe was performed with standard conditions and normalized to *scR1*. Slight difference between *trf4-236* mutant allele and *trf4*Δ [-] was observed; (**b**) RNA from WT, *xrn1*Δ, *trf4*Δ, *xrn1*Δ *trf4*Δ, *rap1-17* and *rat1-1* strains (BY4741 and BY4742) were analyzed by Northern blot using *TERRA* (C probe: 5′-CACCACACCCACACACCACACCCACA-3′; [40]) and *subTERRA* [51] -specific probes; (**c**) 10 µg of RNA were treated with RNaseA and DNase I (60 min at 37 °C and at RT, respectively) loaded on the gel and probed with Y′-specific subtelomeric probe; (**d**) *subTERRA* are transcribed by RNAPII. *xrn1*Δ and *rpb1-1 xrn1*Δ strains were grown at 25 °C and shifted to 37 °C for 2h. RNA samples were extracted at indicated time points and probed with Y' *subTERRA* probe. Controls *ACT1* mRNA, transcribed by RNAPII and *scR1*, transcribed by RNAPIII were probed with specific probes. Experiment was repeated twice; (**e**) 30% of all species of *subTERRA* molecules are polyadenylated. Total RNA was depleted of polyA-RNAs and analyzed by Northern blot with *subTERRA*-specific probe. The efficiency of polyA precipitation was taken into account when calculating polyadenylated fraction.

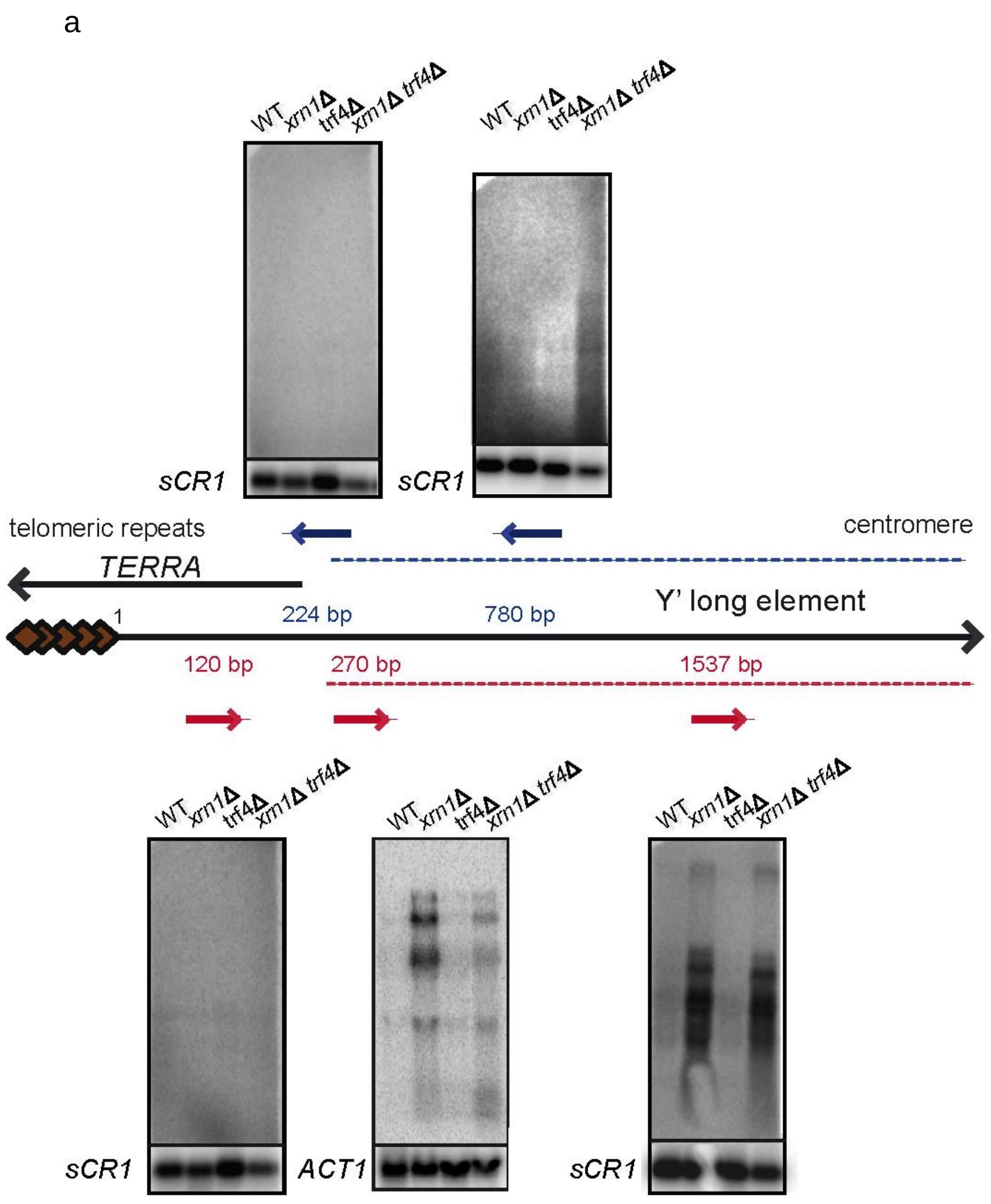

Figure S2, *Cont*.

b

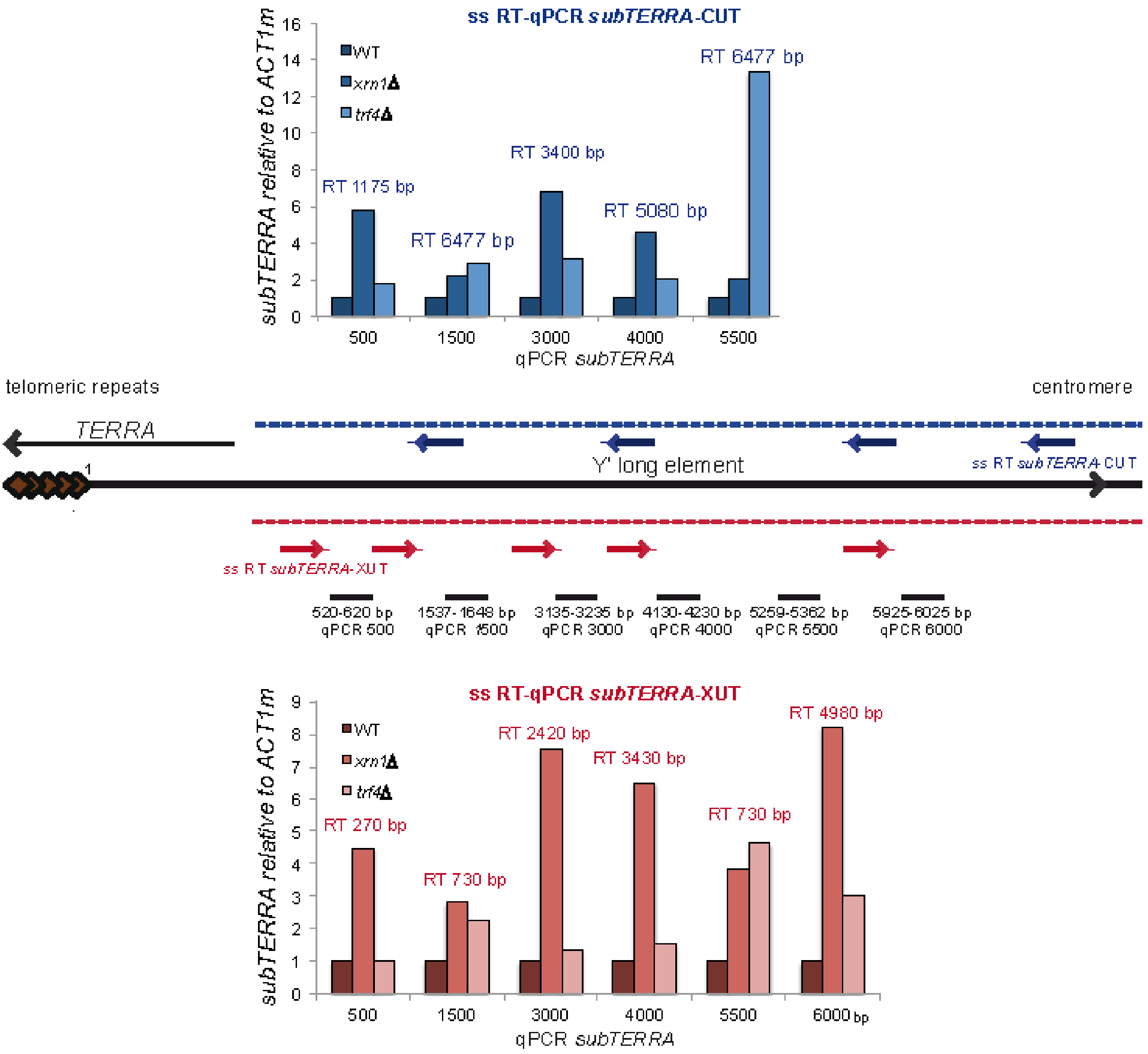

Figure S2, *Cont.*

c

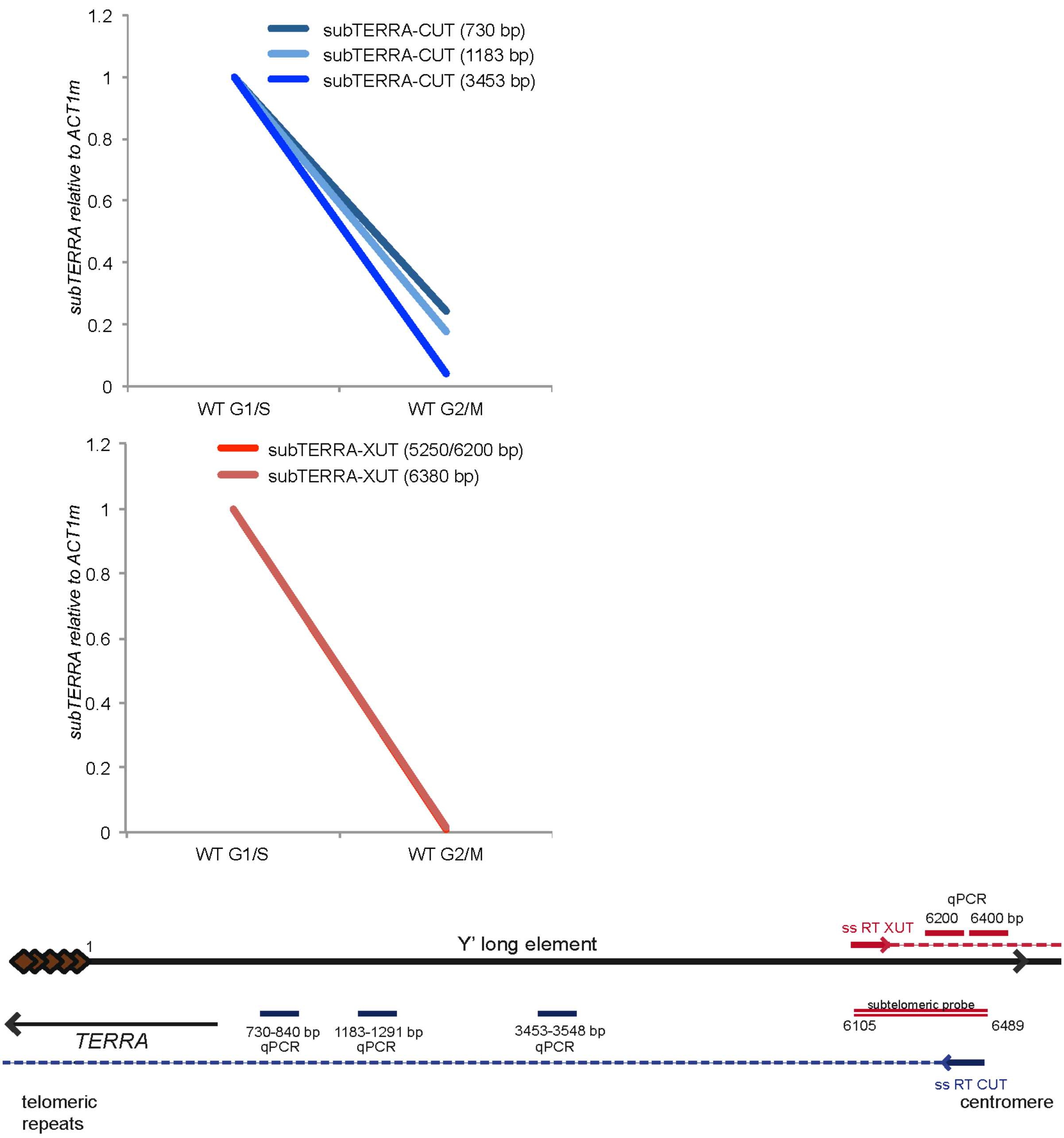

Figure S2. Sense-specific detection of *subTERRA*. (**a**) Northern blot showing specificity of *subTERRA* detection. 10 µg of RNA were loaded on the gel and probed with radiolabelled oligonucleotides (coordinates and position are indicated at schematic view of analyzed region). Probes at the junction of telomere Y' region with no signal detected indicated that *TERRA* and *subTERRA* are discontinuous; (**b**) Sense-specific RT-qPCR. Positions of oligonucleotides used for amplification of *subTERRA* species are shown in red for XUTs and in blue for CUTs. Primers pairs for qPCR are shown in black. Obtained quantities were normalized to *ACT1*m and WT level was set to 1 (biological duplicate); (**c**) Sense-specific RT-qPCR on RNA extracted from cell cycle synchronized WT *bar1*Δ strain (single culture). As above used primers are schematized. qPCR signals were normalized to *ACT1*m and G1/S signals were set as 1.

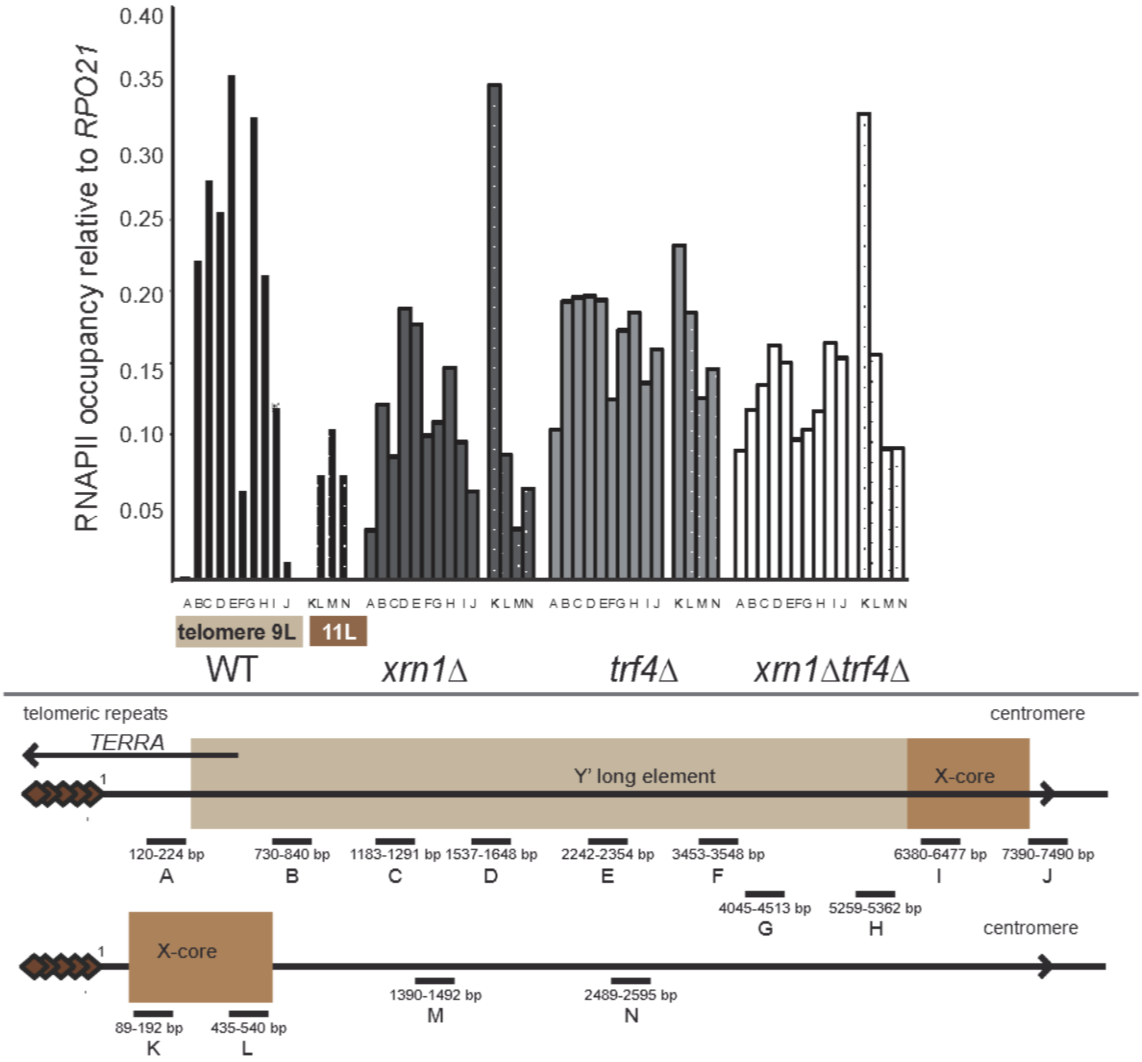

Figure S3. RNAPII occupancy of subtelomeric regions does not increase in G1-synchronized cells. RNAPII-ChIP experiment was performed in *alpha*-factor-synchronized cells grown in YPD. Subtelomeric regions at telomeres 9L (Y') and 11L (only X) were scanned in WT *bar1*Δ, *xrn1*Δ *bar1*Δ, *trf4*Δ *bar1*Δ and *xrn1*Δ *trf4*Δ *bar1*Δ cells (W303). RNAPII occupancy was normalized to levels at *RPO21* locus. Pairs of primers used for qPCR are named with letters and represented on schematic view of analyzed region. Increase of RNAPII occupancy was seen in *xrn1*Δ and *xrn1*Δ *trf4*Δ at one position – K, corresponding to very beginning of subtelomeric region immediately after $TG_{1–3}$ repeats. RNAPII occupancy values are the same or slightly lower when compared to non-synchronized cells.

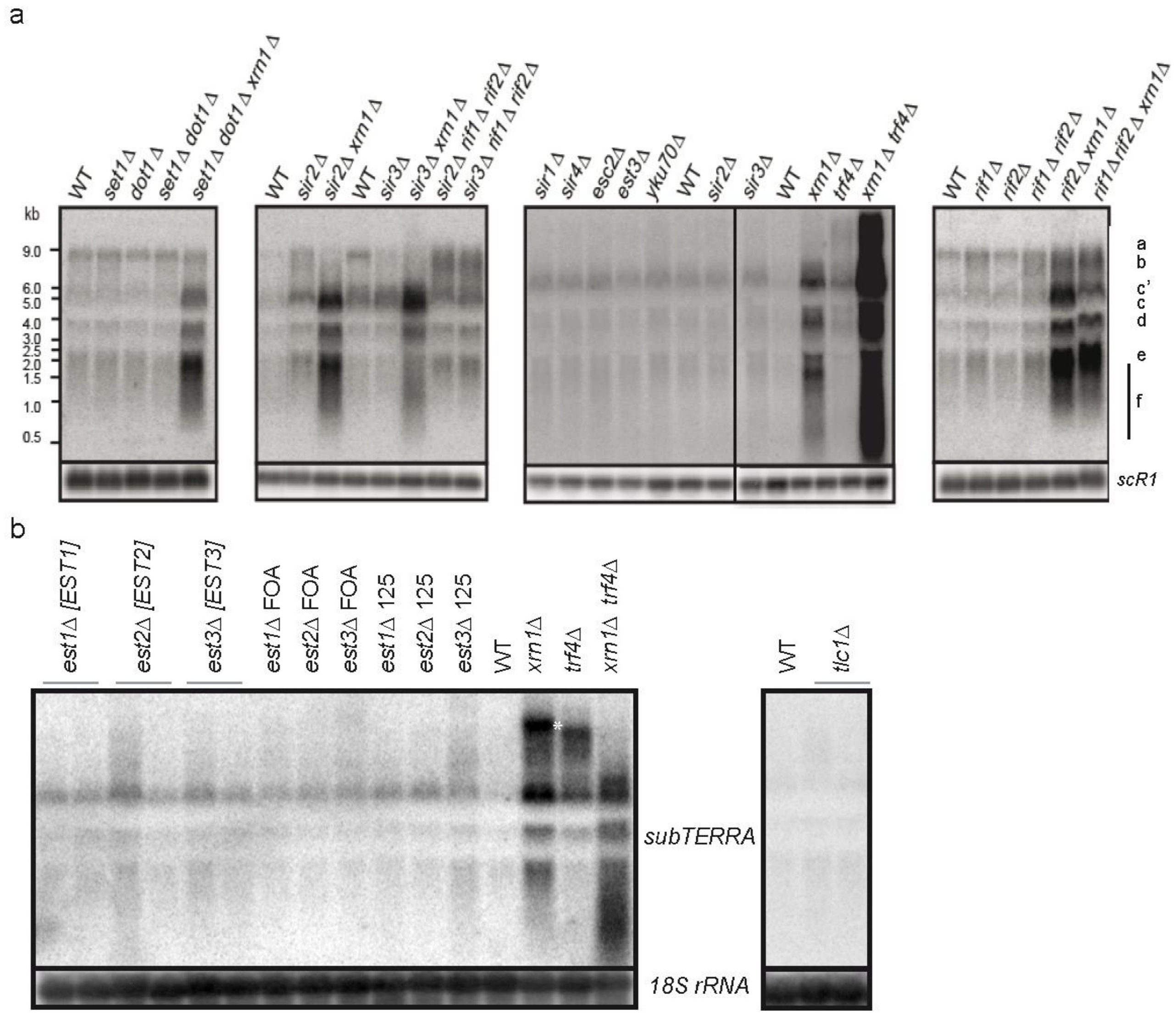

Figure S4. *subTERRA* do not accumulate in single mutants lacking majority of telomere-associated proteins and chromatin modifiers. (**a**) Detection of Y' RNAs in strains mutated for genes implicated in telomere homeostasis and heterochromatinization. *subTERRA* detection and normalization as in Figure 1b, at least biological duplicates were done. Cells were grown in YPD at 30 °C ON to exponential phase; (**b**) No accumulation of *subTERRA* was observed in cells lacking telomerase subunits. Y′ was detected as in (a). Cells were grown in CSM-URA, plasmids were chased on 5-FOA plates and subsequently telomerase negative cells were grown in YPD at 30 °C for 125 generations.

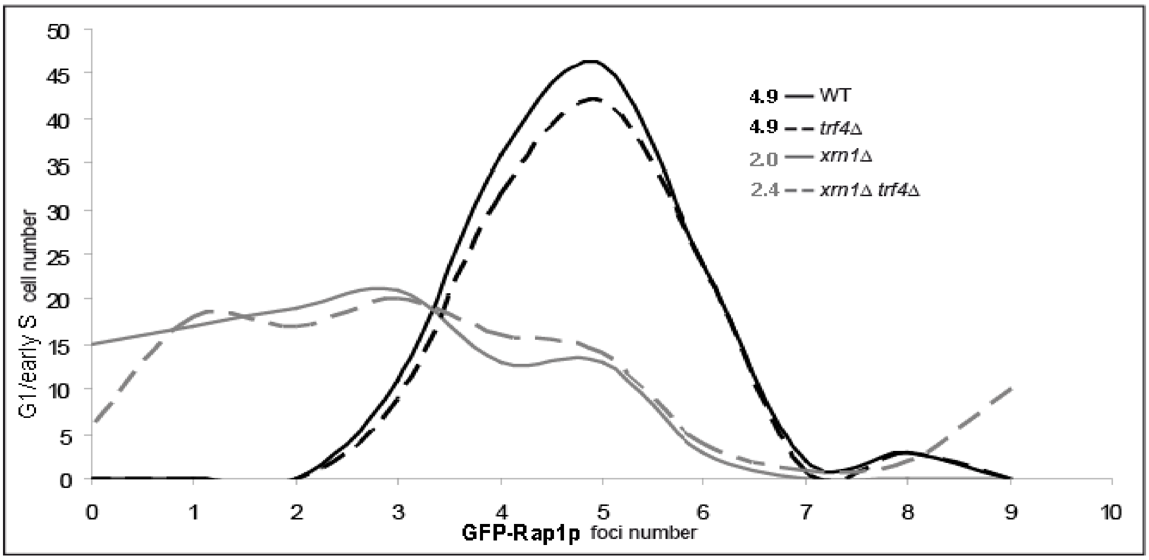

Figure S5. *xrn1*Δ mutation causes decrease in number of telomere clusters in G1 and early S cells. Telomeric foci, GFP-Rap1p *in vivo*, were manually counted in at least 100 not budded (G1)/small-budded (early S) cells in exponentially grown YPD cultures (30 °C). In WT and *trf4*Δ cells the mean foci number is 4.9 and is significantly decreased in *xrn1*Δ nd *xrn1*Δ *trf4*Δ cells (2 and 2.4, respectively).

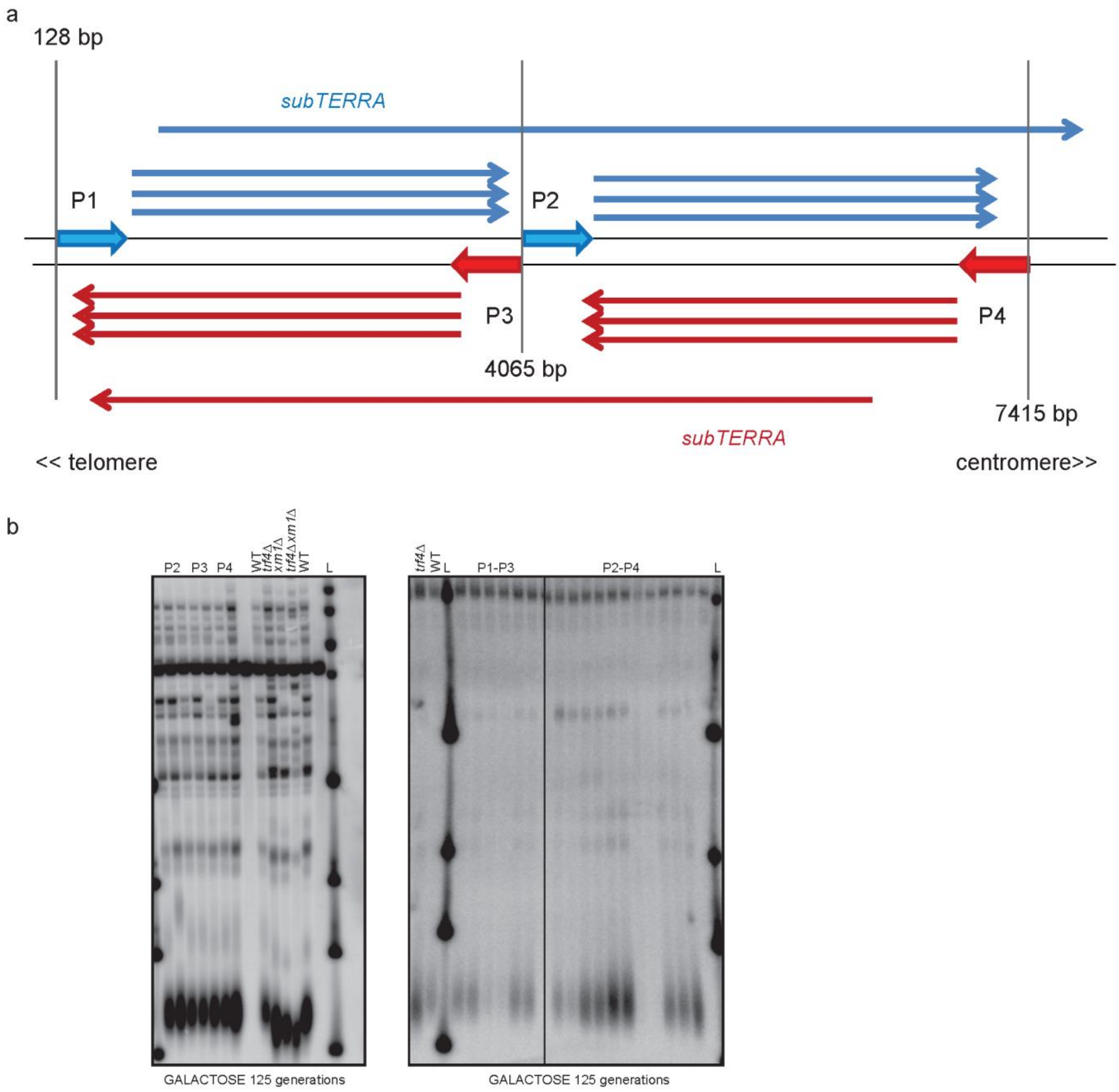

Figure S6. Induced expression of *subTERRA* species does not change telomere length. (**a**) Schema of integration of *pGAL* promoters into subtelomeric region (P1 and P2 towards centromere, expressing *subTERRA*-CUTs and P3 and P4 towards telomere, expressing *subTERRA*-XUTs). Coordinates are the bp distance from the beginning of telomeric repeats; subtelomeric long Y' element is of 6278 bp followed by X element core (195 bp). Induction of *pGAL* promoter occurs immediately after addition of galactose to the growth media. Expressed species of *subTERRA* are color arrows. Their presence was confirmed by RT-qPCR and Northern blot using sense-specific amplifications and probes; (**b**) Genomic DNA from two independent clones digested with *XhoI* and probed with telomere-specific probe, detecting $TG_{1–3}$ repeats. The 1 kb DNA ladder (NEB) was migrated in the first line, and internal migration control is the band of 2.5 kb. Strains were grown in YPGal medium at 30 °C for 125 generations. Left panel shows strains expressing single *subTERRA* (promoters P2, P3 and P4), right panel strains expressing sense and anti-sense pairs (promoters P1/P3 and P2/P4).

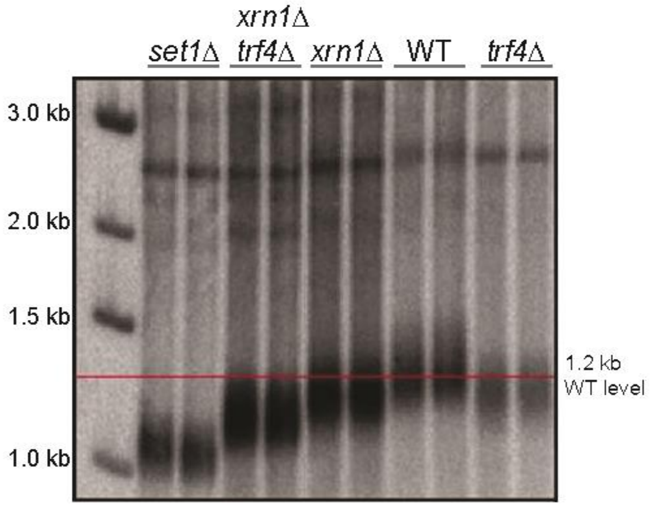

Figure S7. Analysis of telomere length in WT and RNA decay mutants. Genomic DNA from two independent clones of indicated strains in W303 background, digested with *XhoI* and probed with telomere-specific probe, detecting TG_{1-3} repeats. The 1 kb DNA ladder (NEB) was migrated in the first line, and internal migration control is the band of 2.5 kb. Strains were grown in YPD medium overnight at 30 °C. WT length (around 1.2 kb) is a red line.

Table S1. Strains used in this study.

WT	MATa *ade2-1 his3-11,15 leu2-3,112 trp1-1 ura3-1 can1-100*	[106]
*xrn1*Δ	MATa *ade2-1 his3-11,15 leu2-3,112 trp1-1 ura3-1 can1-100 xrn1::ADE2*	[106]
*dcp1*Δ	MATa *ade2-1 his3-11,15 leu2-3,112 trp1-1 ura3-1 can1-100 dcp1::URA3*	[106]
*upf1*Δ	MATa *ade2-1 his3-11,15 leu2-3,112 trp1-1 ura3-1 can1-100 upf1::HIS3*	[106]
*upf2*Δ	MATa *ade2-1 his3-11,15 leu2-3,112 trp1-1 ura3-1 can1-100 nmd2::HIS3*	[106]
*upf3*Δ	MATa *ade2-1 his3-11,15 leu2-3,112 trp1-1 ura3-1 can1-100 upf3::HIS3*	[106]
*rnt1*Δ	MATa *trp1-1 ura3-52 his3-11,15 ade2-1 rnt1::TRP1*	[52]
*ccr4*Δ	MATa *ade2-1 his3-11,15 leu2-3,112 trp1-1 ura3-1 can1-100 ccr4::KanMX*	[44]
*trf5*Δ	MATa *his3Δ1 leu2Δ0 met15Δ0 ura3Δ0 trf5::KanMX*	[107]
*trf4*Δ	MATa *ade2-1 his3-11,15 leu2-3,112 trp1-1 ura3-1 can1-100 trf4::KanMX6*	[44]
*trf4*Δ *xrn1*Δ	MATa *ade2-1 his3-11,15 leu2-3,112 trp1-1 ura3-1 can1-100 xrn1::ADE2 trf4::KanMX6*	[44]
*rrp6*Δ *trf4*Δ	MATalpha *his3Δ1 leu2Δ0 lys2Δ0 ura3Δ0 trf4::HIS rrp6::KanMX*	[107]
*rrp6*Δ	MATalpha *his3Δ1 leu2Δ0 lys2Δ0 ura3Δ0 rrp6::KanMX*	[107]
rap1-17	MATalpha *ade2-1 trp1-1 ura3-1 leu2-3,112 his3-11-15 can1-100 rap1-17 adh4::URA3-TEL rad5-535*	[108]
*rpb1-1 xrn1*Δ	MATalpha *his4-912 lys2-128 leu2Δ1 trp1Δ63 ura3-52 rpb1-1 xrn1::KanMX*	[44]
*set1*Δ	MATa *ade2-1 trp1-1 leu2-3,112 his3-11,15 ura3 can1-100 set1::KanMX*	[44]
*rat1-1 xrn1*Δ	MATa *leu2-1 ura3-52 his3-200 rat1-1 xrn1::URA3*	[52]
rat1-1	MATa *trp- leu2-1 ura3-52 his3-200 rat1-1*	[52]
WT *bar1*Δ	MATa *ade2-1 can1-100 his3-11,5 leu2-3,112 trp1-1 ura3-1 bar1::LEU2*	From D. Libri
*trf4*Δ *bar1*Δ	MATa *ade2-1 can1-100 his3-11,5 leu2-3,112 trp1-1 ura3-1 bar1::LEU2 trf4::KanMX*	This study
*xrn1*Δ *bar1*Δ	MATa *ade2-1 can1-100 his3-11,5 leu2-3,112 trp1-1 ura3-1 bar1::LEU2 xrn1::KanMX*	This study
*xrn1*Δ *trf4*Δ *bar1*Δ	MATa *ade2-1 can1-100 his3-11,5 leu2-3,112 trp1-1 ura3-1 bar1::LEU2 xrn1::KanMX trf4::HIS3*	This study
*sir2*Δ *rif1*Δ *rif2*Δ *bar1*Δ	MATa *ade2–1 trp1–1 leu2–3,112 his3–11,15 ura3 can1–100 bar1Δ rif1::NAT rif2::HphMX sir2::KanMX*	This study
*sir3*Δ *rif1*Δ *rif2*Δ *bar1*Δ	*MATa ade2–1 trp1–1 leu2–3,112 his3–11,15 ura3 can1–100 bar1Δ rif1::NAT rif2::HphMX sir3::KanMX*	This study
*rif1*Δ *bar1*Δ	MATa *ade2-1 trp1-1 leu2-3,112 his3-11,15 ura3 can1-100 bar1Δ rif1::NAT*	From V. Geli
*rif2*Δ *bar1*Δ	MATa *ade2-1 trp1-1 leu2-3,112 his3-11,15 ura3 can1-100 bar1Δ rif2::HphMX*	From V. Geli
*rif1*Δ *rif2*Δ *bar1*Δ	MATa *ade2-1 trp1-1 leu2-3,112 his3-11,15 ura3 can1-100 bar1Δ rif1::NAT rif2::HphMX*	From V. Geli
*xrn1*Δ *rif1*Δ *rif2*Δ *bar1*Δ	MATa *ade2-1 trp1-1 leu2-3,112 his3-11,15 ura3 can1-100 bar1Δ rif1::NAT rif2::HphMX xrn1::KanMX*	This study
*xrn1*Δ *rif2*Δ *bar1*Δ	MATa *ade2-1 trp1-1 leu2-3,112 his3-11,15 ura3 can1-100 bar1Δ rif2::HphMX xrn1::KanMX*	This study
*sir3*Δ *bar1*Δ	MATa *ade2-1 can1-100 his3-11,5 leu2-3,112 trp1-1 ura3-1 bar1::LEU2 sir3::TRP*	This study
*xrn1*Δ *sir3*Δ *bar1*Δ	MATa *ura3-52 leu2-3,112 ade2-1 lys1-1 his5-2 can1-100 sir3::LEU2 xrn1::KanMX*	This study
*sir2*Δ *xrn1*Δ	MATalpha *ade2-1 trp1-1 leu2-3 his3-11,15 ura3-1 can1-100 sir2::TRP1 xrn1::KanMX*	This study
*sir2*Δ	MATalpha *ade2-1 trp1-1 leu2-3 his3-11,15 ura3-1 can1-100 sir2::TRP1*	From Ann Ehrenhofer-Murray

Table S1. *Cont.*

dot1Δ	MATa *ade2-1 trp1-1 leu2-3,112 his3-11,15 ura3 can1-100 dot1::KanMX*	From V. Geli
set1Δ dot1Δ xrn1Δ	MATalpha *ade2-1 trp1-1 leu2-3,112 his3-11,15 ura3 can1-100 set1::URA3 dot1::KanMX xrn1::HIS3*	This study
set1Δ dot1Δ	MATa *ade2-1 trp1-1 leu2-3,112 his3-11,15 ura3 can1-100 set1::URA3 dot1::KanMX*	From V. Geli
WT BY4741	MATa *his3Δ1 leu2Δ0 met15Δ0 ura3Δ0*	Euroscarf collection
trf4Δ	MATa *his3Δ1 leu2Δ0 met15Δ0 ura3Δ0 trf4::HIS3*	[47]
xrn1Δ trf4Δ	MATa *his3Δ1 leu2Δ0 met15Δ0 ura3Δ0 xrn1::KanMX trf4::HIS3*	[47]
xrn1Δ	MATa *his3Δ1 leu2Δ0 met15Δ0 ura3Δ0 xrn1::KanMX4*	Euroscarf collection
sir1Δ	MATa *his3Δ1 leu2Δ0 met15Δ0 ura3Δ0 sir1::KanMX4*	Euroscarf collection
sir4Δ	MATa *his3Δ1 leu2Δ0 met15Δ0 ura3Δ0 sir4::KanMX4*	Euroscarf collection
sir3Δ	MATa *his3Δ1 leu2Δ0 met15Δ0 ura3Δ0 sir3::KanMX4*	Euroscarf collection
sir2Δ	MATa *his3Δ1 leu2Δ0 met15Δ0 ura3Δ0 sir2::KanMX4*	Euroscarf collection
yku70Δ	MATa *his3Δ1 leu2Δ0 met15Δ0 ura3Δ0 yku70::KanMX4*	Euroscarf collection
esc2Δ	MATa *his3Δ1 leu2Δ0 met15Δ0 ura3Δ0 esc2::KanMX4*	Euroscarf collection
est3Δ	MATa *his3Δ1 leu2Δ0 met15Δ0 ura3Δ0 est3::KanMX4*	Euroscarf collection
lsm1Δ	MATa *his3Δ1 leu2Δ0 met15Δ0 ura3Δ0 lsm1::KanMX*	Euroscarf collection
lsm7Δ	MATa *his3Δ1 leu2Δ0 met15Δ0 ura3Δ0 lsm7::KanMX*	Euroscarf collection
pat1Δ	MATa *ade2 arg4 leu2-3,112 trp1-289 ura3-52 pat1::TRP1KL*	[109]
rat1-1	MAT*alpha his3 leu2 met15 ura3 rat1-1::NatMX*	[42]
rat1-1	MATa *his3 leu2 met15 ura3 rat1-1::NatMX*	[42]
rap1-17	MATa *his3 leu2 ura3 rap1-17 can1*	[42]
rap1-17	MATa *his3 leu2 met15 ura3 rap1-17 can1*	[42]
telIXL-URA3	MATa *ura3Δ851 leu2Δ1 his3Δ200 lys2Δ202 URA3 at position 7*	[61]
GFP-Rap1p	MATa *ade2-1::ADE2 trp1-1 leu2-3,112 his3-11,15 ura3 can1-100 GFP-RAP1::LEU2 NUP133-cherry::KanMX*	This study
GFP-Rap1p *xrn1Δ*	MATa *ade2-1 trp1-1 leu2-3,112 his3-11,15 ura3 can1-100 GFP-RAP1::LEU2 xrn1::ADE2 NUP133-cherry::KanMX*	This study
GFP-Rap1p *trf4Δ*	MATa *ade2-1::ADE2 trp1-1 leu2-3,112 his3-11,15 ura3 can1-100 GFP-RAP1::LEU2 trf4::TRP1 NUP133-cherry::KanMX*	This study
GFP-Rap1p *xrn1Δ trf4Δ*	*MATa ade2-1 trp1-1 leu2-3,112 his3-11,15 ura3 can1-100 GFP-RAP1::LEU2 xrn1::ADE2 trf4::TRP1 NUP133-cherry::KanMX*	This study
tlc1Δ	MATalpha *ura3Δ0 leu2Δ0 his3Δ1 met15Δ0 tlc1::TA_Pralpha2_Nat*	From T. Teixeira

Table S1. *Cont.*

est3Δ [*EST3]*	MATa *ura3Δ0 leu2Δ0 his3Δ1 met15Δ est3::KanMX4 [pVL232-EST3-URA3]*	From T. Teixeira
est2Δ [*EST2*]	MATa *ura3Δ0 leu2Δ0 his3Δ1 met15Δ est2::KanMX4 [pVL232-EST2-URA3]*	From T. Teixeira
est1Δ [*EST1*]	MATa *ura3Δ0 leu2Δ0 his3Δ1 met15Δ est1::KanMX4 [pVL232-EST1-URA3]*	From T. Teixeira
pGAL(P2)subTERRA	MATa *ade2-1::ADE2 trp1-1 leu2-3,112 his3-11,15 ura3 can1-100 RAP1::GFP-LEU2 Nup133-cherry::KanMX TRP1::pGAL-RNAtelo2=P2*	This study (3 clones)
pGAL(P3)subTERRA	MATa *ade2-1::ADE2 trp1-1 leu2–3,112 his3-11,15 ura3 can1-100 RAP1::GFP-LEU2 Nup133-cherry::KanMX TRP1::pGAL-RNAtelo5=P3*	This study (3 clones)
pGAL(P4)subTERRA	MATa *ade2-1::ADE2 trp1-1 leu2-3,112 his3-11,15 ura3 can1-100 RAP1::GFP-LEU2 Nup133-cherry::KanMX TRP1::pGAL-RNAtelo8=P4*	This study (3 clones)
pGAL(P1_P3)subTERRA	MATa *ade2-1::ADE2 trp1-1 leu2-3,112 his3-11,15 ura3 can1-100 RAP1::GFP-LEU2 Nup133-cherry::KanMX TRP1::pGAL-RNAtelo5 TEL-1::HIS3*	This study (3 clones)
pGAL(P2_P4)subTERRA	MATa *ade2-1::ADE2 trp1–1 leu2-3,112 his3-11,15 ura3 can1-100 RAP1::GFP-LEU2 Nup133-cherry::KanMX TRP1::pGAL-RNAtelo2 HIS3::pGAL-RNAtelo8*	This study (6 clones)

Table S2. Plasmids used in this study.

WT-*XRN1*	*XRN1;* AmpR; *LEU2*; CEN	[104]
xrn1-E176G	*xrn1-E176G;* AmpR; *LEU2*; CEN	[104]
WT-*TRF4*	*TRF4*; AmpR; *TRP1-HIS3*; CEN	[55]
trf4-236	*trf4-236;* AmpR; *TRP1-HIS3*; CEN	[55]
pRS413	AmpR; *HIS3*; CEN	[105]
pRS315	AmpR; *LEU2*; CEN	[28]
